# PLGA Nanoparticles Co-encapsulating NY-ESO-1 Peptides and IMM60 Induce Robust CD8 and CD4 T Cell and B Cell Responses

**DOI:** 10.3389/fimmu.2021.641703

**Published:** 2021-02-25

**Authors:** Yusuf Dölen, Uzi Gileadi, Ji-Li Chen, Michael Valente, Jeroen H. A. Creemers, Eric A. W. Van Dinther, N. Koen van Riessen, Eliezer Jäger, Martin Hruby, Vincenzo Cerundolo, Mustafa Diken, Carl G. Figdor, I. Jolanda M. de Vries

**Affiliations:** ^1^Department of Tumor Immunology, Radboud University Medical Center, Radboud Institute for Molecular Life Sciences, Nijmegen, Netherlands; ^2^Oncode Institute, Nijmegen, Netherlands; ^3^Medical Research Council Human Immunology Unit, Radcliffe Department of Medicine, Weatherall Institute of Molecular Medicine, University of Oxford, Oxford, United Kingdom; ^4^Aix Marseille Univ, CNRS, INSERM, CIML, Centre d'Immunologie de Marseille-Luminy, Marseille, France; ^5^Institute of Macromolecular Chemistry v. v. i., Academy of Sciences of the Czech Republic, Prague, Czechia; ^6^TRON - Translational Oncology at the University Medical Center of the Johannes Gutenberg University Mainz gGmbH, Mainz, Germany

**Keywords:** NY-ESO-1, iNKT cell, B cell epitope, peptide vaccine, IMM60, PLGA nanoparticle, CD8 T cell, CD4 T cell

## Abstract

Tumor-specific neoantigens can be highly immunogenic, but their identification for each patient and the production of personalized cancer vaccines can be time-consuming and prohibitively expensive. In contrast, tumor-associated antigens are widely expressed and suitable as an off the shelf immunotherapy. Here, we developed a PLGA-based nanoparticle vaccine that contains both the immunogenic cancer germline antigen NY-ESO-1 and an α-GalCer analog IMM60, as a novel iNKT cell agonist and dendritic cell transactivator. Three peptide sequences (85–111, 117–143, and 157–165) derived from immunodominant regions of NY-ESO-1 were selected. These peptides have a wide HLA coverage and were efficiently processed and presented by dendritic cells *via* various HLA subtypes. Co-delivery of IMM60 enhanced CD4 and CD8 T cell responses and antibody levels against NY-ESO-1 *in vivo*. Moreover, the nanoparticles have negligible systemic toxicity in high doses, and they could be produced according to GMP guidelines. Together, we demonstrated the feasibility of producing a PLGA-based nanovaccine containing immunogenic peptides and an iNKT cell agonist, that is activating DCs to induce antigen-specific T cell responses.

## Introduction

Recent advancements in cancer immunotherapy such as checkpoint blockade therapy, CAR T cells, and neo-epitope-based RNA vaccines show that once activated, the adaptive immune system is capable of recognizing and eradicating tumor cells (1). This recognition is based on tumor antigens which are either normal proteins that are aberrantly or over-expressed in tumors [tumor-associated antigens (TAA)], or proteins that are mutated during tumorigenesis (tumor-specific antigens or neoantigens). While neoantigens are patient-specific, TAAs are widely expressed in a majority of cancer patients with various types of tumors (1). One subset of TAAs is cancer germline antigens which are generally expressed in immune-privileged sites such as germ cells of the testes, in fetal ovaries, and on trophoblasts (1, 2). The immune system is thought not to be desensitized against cancer germline- and mutated-proteins and therefore, T cell responses can be induced against these antigens (3, 4).

Invariant Natural Killer T (iNKT) cells represent a specialized subset of immune cells characterized by the expression of a restricted αβ T cell antigen receptor (TCR) that specifically recognizes lipid antigens such as α-GalCer presented by CD1d molecules expressed by dendritic cells (DCs), macrophages and B cells (5). Upon activation, they rapidly secrete large amounts of cytokines and induce subsequent activation of different cell types, including DCs, NK cells, and T cells (6). Due to the production of cytokines (i.e., IFN-γ, IL-4) and DC activation through CD40-CD40L interaction, iNKT cells can act as a helper T cell to boost cytotoxic T cell (CTL) responses. Hence, α-galactosylceramide (α-GalCer) and its analogs are explored as vaccine adjuvants (7).

NY-ESO-1 antigen has been used in clinical vaccination studies, mainly because of its expression in a broad range of cancers with high incidence (one-third to one-fourth of melanoma, lung, breast, esophageal, liver, gastric, prostate, ovarian, and bladder cancer) (8–10). Vaccinations led to an enhancement of humoral and cellular immune responses and clinical improvements have been documented in some patients, supporting the role of NY-ESO-1 as an attractive antigen for therapeutic vaccination (3, 11, 12). Moreover, the development of potent vaccines with multiple shared tumor antigens including NY-ESO-1 in combination with checkpoint blockade therapy has recently been shown to enhance the clinical response rates of cancer patients (13).

Effective cancer vaccines should induce strong and long-lasting immune responses. Our previous preclinical data with nanoparticle-encapsulated iNKT cell agonists (α- GalCer and IMM60) and antigens (ovalbumin and HPV-E7) in mice demonstrated regression of tumors after a single injection of nanoparticles (14, 15). We observed that iNKT cell agonists have a high adjuvant effect at dosages that could be loaded within PLGA nanoparticles and therefore, has an advantage over TLR ligands that could not be sufficiently loaded (14). Additionally, activation of iNKT cells by PLGA nanoparticles led to activation and mobilization of multiple cell types such as NK cells, B cells, CD4, and CD8 T cells as well as alternative cognate licensing of DCs (15, 16). Moreover, we and others showed that antigen vaccinations, together with iNKT cell agonists, provide a strong immune response and long-lasting tumor regression if employed in combination with checkpoint blockade therapy (15, 17).

Here we describe the design of a PLGA-based nanoparticle, for co-delivery of NY-ESO-1 peptides with an iNKT cell agonist, as a cancer vaccine. We selected IMM60 as an iNKT cell agonist because of its enhanced ability to activate human iNKT-cells compared with α-GalCer, resulting in extended iNKT responses (18). Using NY-ESO-1-specific TCR mRNA transfection of healthy donor T cells, and patient-derived T cells, we confirmed previous observations that peptides are indeed more immunogenic than the whole protein (19) and that the selected peptides could be efficiently processed and presented by multiple HLA types either in solution or in nanoparticle-encapsulated form. Furthermore, we demonstrated enhanced *in vivo* immune responses with nanoparticles compared to soluble peptide injections and a further enhancement due to co-delivery of iNKT cell agonist IMM60 within the nanoparticles.

## Methods

### Reagents

PLGA (Resomer RG 502 H, lactide/glycolide molar ratio 50:50) was purchased from Evonik Solvents. Dichloromethane was obtained from Merck. CryoSure-DMSO from WAK-Chemie. Polyvinyl alcohol 80% (PVA) from Sigma. Pure water from Braun. Isopropyl alcohol, ≥ 99.7% ACN, ≥ 99.9%, MeOH, ≥ 99.9%, and (CHCl_3_, ≥ 99%) were obtained from Sigma-Aldrich. NY-ESO-1 derived peptides; 85–111 (SRLLEFYLAMPFATPMEAELARRSLAQ), 117–143 (PVPGVLLKEFTVSGNILTIRLTAADHR), and 157–165 (SLLMWITQC) were custom synthesized by Genscript and Pichem; 153–167 (LQQLSLLMWITQCFL), and 97–111 (ATPMEAELARRSLAQ) was produced by Genscript. All peptides had >95% purity and concentrations were based on net peptide weights determined by nitrogen analysis. IMM-60 was provided by Ian Walters of iOx Therapeutics. RPMI 1,640 medium was obtained from Life Technologies. Full-length NY-ESO-1 protein was produced in *Escherichia coli* by the Ludwig Institute for Cancer Research, New York branch. 0.5 mg NY-ESO-1 protein was dissolved in 1 ml water containing 240 mg urea, 3.75 mg glycine, 13.8 mg Sodium Dihydrogen Phosphate Monohydrate, and 8.5 mg Sodium Chloride.

### Nanoparticle production

All PLGA nanoparticles (NPs) were prepared using a single emulsion and solvent evaporation–extraction method, as described previously (10). Briefly, 100 mg of PLGA was dissolved in 3 ml of dichloromethane containing 1 mg of each peptide (Supplementary Table 3) and 150 μg IMM60 dissolved in DMSO. This organic phase was added dropwise to 25 ml of aqueous phase containing 2.5% PVA and emulsified for 120 s using a digital probe sonicator (Branson Ultrasonics, Danbury, CT). The organic phase was evaporated overnight at RT while stirring, and nanoparticles were collected by centrifugation at 10,000 rpm (13304 RCF) for 35 min, washed three times with pure water, and lyophilized. Different peptide and IMM60 concentrations were examined and reported in the results section.

### Nanoparticle Characterization

The size and polydispersity index of the nanoparticles was analyzed by dynamic light scattering using a Nanotrac Flex (Microtrac). The peptide content of the NPs was determined by HPLC analysis using a standard dilution of peptides based on net peptide content (15). All amounts of PLGA-NPs used in this study were calculated according to their net peptide contents except for the particles containing full NY-ESO-1 protein which the content could not be determined due to high amounts of urea and glycine contamination. IMM60 content of the NPs was determined by a Corona Veo Charged Aerosol Detector (CAD) coupled to a DIONEX UltiMate 3000 HPLC system (Thermo Fischer Scientific). The NPs were dissolved in DMSO for a complete dissolution of the components and analyzed by CAD on an XSelect CSH C_18_ 2.5 μm 3.0 × 150 mm XP column (Waters) with VanGuard Cartridges (Waters) coupled to a column heater (65°C), eluents MeOH-Formic Acid-Triethylamine (99.0/0.05/0.05 vol. %) with isocratic gradient flow rate = 1.0 ml·min^−1^. The quantity of IMM60 was calculated by interpolation of the standard calibration curves of IMM60 performed in the same way as for the NPs. The endotoxin content of the nanoparticles was analyzed using the gel-clot method by Eurofins PROXY laboratories, Leiden, The Netherlands, and found to be lower than 0.1 EU/mg particles.

### *In vitro* Antigen Presentation With TCR mRNA Transfected T Cells

HLA typed leukapheresis products were obtained from the blood bank Sanquin, Nijmegen, and subjected to density gradient separation with Ficoll to obtain PBMCs. Monocytes were isolated from PBMCs *via* positive MACS separation with CD14 microbeads according to the manufacturer's protocol (Miltenyi Biotech). The remaining cells were subjected to the untouched separation of T cells with either the CD8 T Cell Isolation Kit or the CD4 T Cell Isolation Kit according to the manufacturer's protocol (Miltenyi Biotech). Monocytes, CD8, or CD4 T cells were frozen in FBS 10% DMSO solution and stored in liquid nitrogen until further use. Monocytes were thawed, and 10 × 10^6^ cells were cultured at 37°C 5% CO_2_ in 8 ml of full RPMI medium (supplemented with 10% FBS, 100 U/ml penicillin, 100 μg/ml streptomycin, 2 mM ultraglutamine) containing 300 U/ml IL-4 and 450 U/ml GM-CSF to generate DCs. On day 3, 3600 U IL-4 and 5400 U GM-CSF were added. On day 6, floating immature DCs were harvested, and 20 × 10^3^ cells/well were plated in a 96 U-bottom plate. Peptides dissolved in DMSO (free form) or encapsulated within nanoparticles were prepared in different concentrations based on net peptide contents in full RPMI and added to the wells. DCs were cultured with antigens for 24 h at 37°C 5% CO_2_. Autologous T cells isolated from the PBMCs were thawed, transferred to full RPMI medium containing 2 μg/ml DNase and incubated for 30 min at 37°C 5% CO_2_. 10 ml of PBS was added, and the cell suspension was centrifuged (8 min, 300 g at room temperature). T cells were counted, 10 ml of serum-free *X-VIVO* 15 medium (Lonza) was added, and the cell suspension was centrifuged for 8 min, 300 g at room temperature. Cells were resuspended in serum-free *X-VIVO* 15 medium in a concentration of 40 × 10^6^ cells/ml and transferred to 4 mm gap Gene Pulser/MicroPulser Electroporation Cuvettes (Bio-Rad). Ten Microgram of mRNA encoding α and β chains of TCR recognizing NY-ESO-1 was mixed with the cells and electroporated with a single square pulse of 500 V for 3 ms using a Gene Pulser Xcell Electroporation System (Bio-Rad). Electroporated cells were transferred to a tube containing 1 ml IVS medium (IMDM GlutaMAX + 5% human AB serum) and incubated for 2 h at 37°C 5% CO_2_. 50 × 10^3^ TCR transfected T cells were added to each well of DCs. LPS was added on the wells to a final concentration of 0.2 ug/ml. Supernatants were collected 72 h after the establishment of DC-T cell co-cultures and analyzed for IFN-γ by ELISA.

### *In vitro* Antigen Presentation Assay With Patient-Derived PBMCs

Frozen PBMCs of two patients who participated in a clinical vaccination trial using NY-ESO-1 whole protein in ISCOMATRIX followed by a booster with recombinant fowlpox virus expressing NY-ESO-1 (20) were analyzed for a response to NY-ESO-1 derived peptides. These PBMCs were previously stimulated for 14 days *in vitro* with a pool of overlapping peptides covering the entire sequence of NY-ESO-1 protein. EBV-transformed B cells of the same patients were loaded with peptides dissolved in DMSO (free form) or nanoparticles for 18 h. These were then used as antigen-presenting cells in co-culture with autologous PBMCs. Separately, free form peptides and nanoparticles were incubated with PBMCs without being loaded to EBV transformed B cells. 5 h later, CD8 and CD4 T cells in the co-cultures were analyzed for intracellular IFN-γ by flow cytometry. For stimulation with NY-ESO-1_157−165_, HLA-A^*^02:01 positive EBV-transformed B cells were loaded with the peptide and then co-cultured with a T-cell clone (4D8) which specifically recognizes this peptide. Intracellular IFN-γ production was assessed by flow cytometry.

### Mice and Tissues

Wild-type C57BL/6J were obtained from Charles River, Germany. AAD mice [Immp2l^Tg(HLA−A/H2−D)2Enge^] were obtained from Jackson, USA. HHD mice were bred in the animal facility of the University of Oxford. All mice were aged between 8 and 15 weeks at the start of experiments. 1G4-HHD mice are transgenic for a mouse-human hybrid TCR m1G4 (with the human variable domains of the human 1G4 TCR that is specific for NY-ESO-1_157−165_ peptide/HLA-A2 complex). All mice were maintained under specific pathogen-free conditions at the Central Animal Laboratory of Radboudumc (Nijmegen, The Netherlands, or at the animal facility of the University of Oxford, UK). Drinking water and food were provided *ad libitum*. PLGA nanoparticles were dissolved in ice-cold PBS by vortexing for 30 s before injected *via* iv route at the tail vein. Blood was collected *via* tail vein puncture or retro-orbital puncture during terminal anesthesia. Spleens were isolated under sterile conditions and stored at 4°C in RPMI 1640 medium supplemented with 100 U/ml penicillin and 100 μg/ml streptomycin until processing for maximally 2 h. Spleens were meshed through a 100 μm cell strainer by using a syringe plunger. The cell suspension was spun at 400 × g for 5 min and resuspended in 3 ml of 1x ammonium chloride solution for the lysis of erythrocytes. After 5 min of incubation at room temperature, cells were washed with 10 ml of PBS. Cells were counted by a hemocytometer and cultured in full RPMI 1,640 medium supplemented with 50 μM 2-mercaptoethanol in 96 well plates.

### *In vivo* Priming

F1 (HHD × C57BL/6J) mice were intravenously injected with 1.1 ug (0.6 nmol) peptide-3-LP (153–167, LQQLSLLMWITQCFL) either in solution or encapsulated within nanoparticles mixed with 50 ng IMM60 in solution. Twenty eight days later all mice were boosted with iv injection of 1.1 ug peptide-3-LP in a solution mixed with 50 ng IMM60. Eight days after the boost, mice were euthanized, splenocytes were processed and stained with fluorescent antibodies and tetrameric pMHC (HLA-A2/K^b^ NY-ESO-1_157−165_) (21) to identify antigen-specific CD8 T cell frequencies. F1 (HHD × B6SJLCD45.1) mice were intravenously injected with nanoparticles containing 2,000 ng (1.1 nmol), 100 ng (55 pmol), or 10 ng (5.5 pmol) peptide-3-LP intravenously and 10 days later, mice were euthanized, splenocytes processed, and stained using fluorescent antibodies and tetrameric pMHC (HLA-A2/K^b^ NY-ESO-1_157−165_). Cells were analyzed on a BD LSR Fortesa flow cytometer.

C57Bl/6 mice were intravenously injected with 1 mg NPs containing all three peptides (8.8 ug Peptide-1, 5.9 ug Peptide-2, and 12 ug Peptide-3 per mg NP) with 1.2 ug IMM60 per mg NP or (6.1 ug Peptide-1, 4.3 ug Peptide-2, and 11 ug Peptide-3 per mg NP) without IMM60 (last two rows of Supplementary Table 3B). Seven days later, splenocytes were isolated and frozen in FBS 10% DMSO. Once thawed, splenocytes were stimulated *ex vivo* with 10 uM peptides 1, 2, and 3 separately. Fourty eight hours later, IFN-γ levels in culture supernatants were determined by ELISA. Splenocytes were stimulated for 16 h at 37°C/5% CO_2_ before the addition of BFA (Sigma) (10 μg/ml) and cultured for 4 h. Cells were washed and surface stained with CD3, CD8, CD4, and LIVE/DEAD cell stain (Invitrogen, UK). Subsequently, the cells were washed, treated with Cytofix and Perm Wash (BD biosciences) according to the manufacturer's instructions, and stained with IFN-γ-PE (BD biosciences) intracellularly.

### *In vivo* Cytotoxicity Assay

Groups of homozygous AAD mice were intravenously injected with nanoparticles encapsulating all three peptides with or without IMM60 and nanoparticles only encapsulating IMM60 and peptide-3. All groups were dosed based on 6 ug peptide-3 content. Injections were repeated 28 days later or the experiment proceeded to the next step after the first injections. Six days after the injection, naive AAD mice splenocytes were loaded either with peptide-3, or HPV (irrelevant) peptide and stained with 5 uM celltrace violet, and celltrace CFSE, respectively. Antigen-loaded splenocytes were transferred as target cells to mice vaccinated with nanoparticles. One day later, all mice were euthanized, and cytotoxicity in spleens was measured by flow cytometry using the following formula:

Ratio = Irrelevant Percentage: Relevant Percentage

Percent Specific killing = [1–(non-vaccinated control ratio/Experimental ratio)] × 100

### *In vivo* Tumor Challenge

A group of homozygous AAD mice was vaccinated intravenously with 1 mg NP (peptide mix+IMM60) containing 11.4 ug peptide-1, 13.4 ug peptide-2, and 11.5 ug peptide-3, and 1.39 ug IMM60. Seven months later groups of vaccinated and non-vaccinated mice were inoculated with 2.5 × 10^5^ 1C12 sarcoma cells expressing full-length NY-ESO-1. 1C12, a Methyl Colanthrene (MCA)-induced murine tumor cell line, was transduced with a NY-ESO-1 expressing lentiviral vector and cloned by limiting dilution. The parental MCA-induced cell line was isolated from a tumor that emerged in MCA injected (intramuscular) HHD mice. Nine days after tumor inoculation, the vaccinated group received a booster dose and tumor growths were recorded. Mice that rejected the first tumors were re-challenged with 5 × 10^5^ 1C12 sarcoma cells on day 74. On day 109, all mice were euthanized, spleens and blood were isolated. Splenocytes were *ex-vivo* restimulated with different peptides, and supernatants were collected 72 h later for IFN-γ analysis.

### *In vivo* Toxicology

A single-species preclinical toxicology study was performed by Charles River Laboratories (Edinburg, UK) to determine the potential toxicity of a single intravenous (bolus) injection of PLGA nanoparticles containing 8.8 ug Peptide-1, 5.9 ug Peptide-2, and 12 ug Peptide-3 with 1.2 ug IMM60 per mg NP or 6.1 ug Peptide-1, 4.3 ug Peptide-2 and 11 ug Peptide-3 per mg NP without IMM60 in mice. One control group received only formulation buffer, one control group received 50 mg/kg PLGA particles containing NY-ESO-1 peptides (SRLLEFYLAMPFATPMEAELARRSLAQ, PVPGVLLKEFTVSGNILTIRLTAADHR, SLLMWITQC), and three groups received three dose levels (0.5, 5, and 50 mg/kg) of PLGA particles containing IMM60 and NY-ESO-1 peptides (SRLLEFYLAMPFATPMEAELARRSLAQ, PVPGVLLKEFTVSGNILTIRLTAADHR, SLLMWITQC). Fifty mice were assigned to terminal necropsy 1 day after injection to monitor toxicological parameters at the peak level. Thirty mice were subjected to necropsy 7 days later to monitor intercurrent mortality and recovery of initial findings. Blood was collected, transferred into tubes containing lithium heparin, and processed for plasma, which was analyzed for Alanine aminotransferase (ALT), Aspartate aminotransferase (AST), and Alkaline phosphatase (ALP). Essential organs such as the liver, spleen, lungs, kidneys, heart, bone marrow, and thymus were stored in fixative. Tissues were processed at Charles River Edinburgh Ltd. PROPATH. Microscopic evaluation was conducted by a senior veterinary pathologist on all tissues.

### ELISA

Human and mouse IFN gamma uncoated ELISA Kits (Invitrogen) were used to determine levels of IFN-γ in serum or culture media according to product protocols. Serum samples were diluted 1/8 −1/10 in blocking buffer before adding to ELISA plates.

For NY-ESO-1 specific antibody detection, ELISA plates were coated by overnight incubation with 10 μg/ml NY-ESO-1 protein or 30 μg/ml NY-ESO-1 protein and 30 μg/ml of each peptide in 100 ul PBS, separately. After washing with wash buffer (PBS, 0.5% Tween-20) plates were incubated with Solution B - Blocking Buffer (Mouse Anti-OVA IgG1 Antibody ELISA Kit 30,105, Chondrex) for 1 h. Sera were diluted 1/100 or 1/500, added into wells, and incubated for 2 h. The plates were incubated for 1 h with horseradish peroxidase-conjugated anti-mouse IgG1 (30133) or IgG2c (30293) (Chondrex) followed by the addition of tetramethylbenzidine substrate solution (Chondrex). The reaction was stopped by the addition of 2N H_2_SO_4_, and the absorbance was read at 450 nm.

### Statistical Analysis

Levene's test was used to assess the homogeneity of group variances. An unpaired *t*-test was used to compare the means of two groups. For comparing more than two groups, one-way ANOVA or Kruskal-Wallis test was used with Tukey's or Dunn's *post-hoc* tests. GraphPad Prism version 9.0.0 for Windows was used for all statistical analysis and figures. ^*^P < 0.05; ^**^P < 0.01; ^***^P < 0.001; and ^****^P < 0.0001. ns, not significant. Mice were randomly assigned to all experimental groups based on online randomization software.

## Results

### Peptides Derived From NY-ESO-1 Are Functional in PLGA Nanoparticles

The long NY-ESO-1 peptides that were used in this study contain known NY-ESO-1-derived peptide epitopes in the context of their respective HLA alleles (peptides-1 and−2 in Table 1) and are therefore, suitable candidates for the final nanoparticle formulation (20, 22, 23). To increase the coverage and immunogenicity of the final product, the short peptide 157–165 (peptide-3 in Table 1) was also included. This third peptide was preferred at minimal length to avoid the generation of an adjoining cryptic epitope present in the long peptide 153–167 (peptide-3-LP) with more dominant immunogenicity (24). Together, the selected peptides cover more than 80% of the European population for both class-I and class-II HLA alleles and are therefore, suitable candidates for inclusion into a nanoparticle cancer vaccine (Supplementary Table 1) (25).

**Table 1:** Epitopes, their binding HLA allele types, and positions in the NY-ESO-1 protein sequence.

**Immunogenic epitopes of NY-ESO-1**				
**HLA**	**Peptide sequence**	**Position**	**Peptide position**	**Long peptide sequence**
DR15	AGATGGRGPRGAGA	37–50		
A*31:01	ASGPGGGAPR	53–62		
B*07:02	APRGPHGGAASGL	60–72		
Cw*06:02	ARGPESRLL	80–88		
DRB5*02:02	SRLLEFYLAMPFATP	85–99		
DR2	RLLEFYLAMPFA	86–97		
DRB1*0901	LLEFYLAMPFATPM	87–100		
DPB1*0401/0402	LLEFYLAMPFATPMEAELARRSLAQ	87–111		
DRB1*0101	LLEFYLAMPFATPMEAELARRSLAQ	87–111		
DRB1*04:01	LLEFYLAMPFATPMEAELARRSLAQ	87–111		
DRB1*07:01	LLEFYLAMPFATPMEAELARRSLAQ	87–111		
DR1	EFYLAMPFATPM	89–100		
A*24:02	YLAMPFATPME	91–101	85–111 27 a.a	Peptide-1SRLLEFYLAMPFATPMEAELARRSLAQ
C*03:03/03:04	LAMPFATPM	92–100		
B*35:08:01	LAMPFATPM	92–100		
A*68:01:01	AMPFATPMEAELARR	93–107		
B*51:01	MPFATPMEA	94–102		
B*35:01	MPFATPMEAEL	94–104		
DQB1*0401	PFATPMEAELARR	95–107		
B*52 01	FATPMEAEL	96–104		
C*12:02	FATPMEAELAR	96–106		
B*07:02:01	ATPMEAELARRSLAQ	97–111		
DRB1*04:01/11:01/16:01	PVPGVLLKEFTVSGNILTIRLTA	117–139	117–143 27 a.a	Peptide-2PVPGVLLKEFTVSGNILTIRLTAADHR
DRB1*0401	PGVLLKEFTVSGNILTIRLT	119–138		
DRB1*0101	PGVLLKEFTVSGNILTIRLTAADHR	119–143		
DR7	PGVLLKEFTVSGNILTIRLTAADHR	119–143		
DRB1*04:01	VLLKEFTVSG	121–130		
DR52bramyaDRB1*04:01	LKEFTVSGNILTIRL	123–137		
DRB1*0803	KEFTVSGNILT	124–134		
B*49:01	KEFTVSGNILTI	124–135		
A*68:01	TVSGNILTIR	127–136		
DR4	AADHRQLQLSISSCLQQL	139–156		
A*02:01	SLLMWITQC	157–165	157–165 9 a.a	Peptide-3SLLMWITQC
DPB1*04:01/04:02	SLLMWITQCFLPVF	157–170		

*HLA class-I binding epitopes are in black and HLA class II-binding epitopes are in blue. The long peptide sequences present in nanoparticles are designated as peptide-1, 2, and 3. HLA, human leukocyte antigen; NY-ESO-1, New York esophageal squamous cell carcinoma 1*.

**Figure 1:**
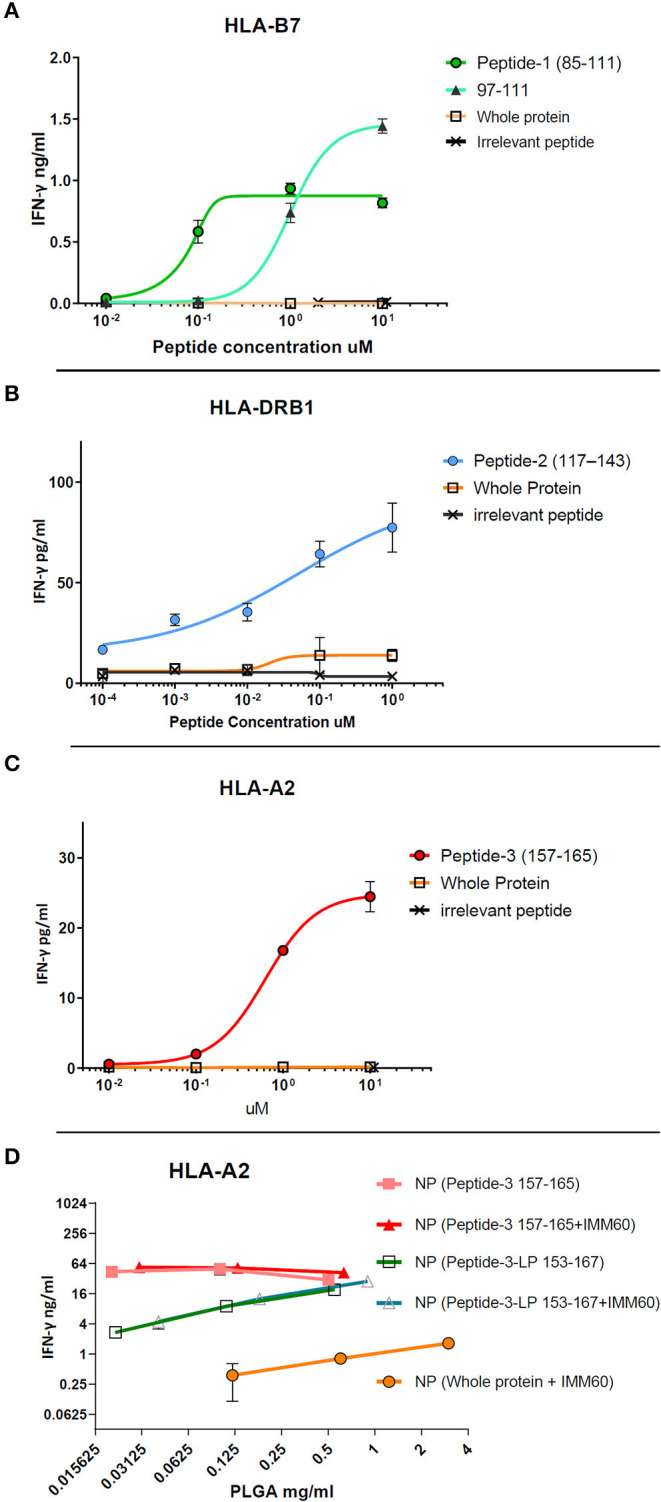
Dose-response curves of selected peptides (dissolved in DMSO) obtained by TCR transfected T cell cultures *in vitro*. **(A)** CD8+ T cell response against free peptide-1 (circle) presented by HLA-B7 is compared with the minimal epitope 97–111 (triangle), the free form whole NY-ESO-1 protein (square), and an irrelevant peptide (cross). **(B)** CD4+ T cell response against peptide-2 (circle) presented by HLA-DRB1 is compared with the free form whole NY-ESO-1 protein (square) and an irrelevant peptide (cross). **(C)** CD8+ T cell response against peptide-3 (circle) presented by HLA-A2 is compared with the free form whole NY-ESO-1 protein (square) and an irrelevant peptide (cross). **(D)** CD8+ T cell response against 157–165 epitope presented by HLA-A2. Nanoparticles containing peptide-3 (157–165) (filled square and triangle) or peptide-3-LP (153–167) (empty square and triangle) with/without IMM60 are compared with nanoparticles containing the whole NY-ESO-1 protein and IMM60 (filled circle) by total PLGA nanoparticle concentration due to the inability to calculate encapsulation of whole NY-ESO-1 protein. Each assay is repeated at least twice with similar results, response curves are fitted by four or five parameter non-linear regression analyses. Mean values are shown with SD.

The free peptides (dissolved in DMSO) were tested for processing and presentation by monocyte-derived DCs (moDCs) of healthy donors to autologous T cells transfected with TCRs derived from NY-ESO-1 specific T cells (26, 27). For peptide-1 (85–111), CD8 T cells were transfected with TCR mRNA (NY#12) recognizing 97–111 peptide of NY-ESO-1 presented by HLA-B^*^0702 (Table 1, Supplementary Table 2A) (27). IFN-γ production was observed after stimulation with peptide-1 (85–111) albeit lower than after stimulation with minimal epitope 97–111 at the highest dose (Figure 1A). To test peptide-2 (117–143), CD4 T cells were transfected with TCR mRNA (NY#3) recognizing 117–139 sequence presented by HLA-DRB1^*^0101 (Table 1, Supplementary Table 2A) (27). IFN-γ production was observed by CD4 T cells in a dose-dependent manner (Figure 1B). To test peptide-3, TCR transfected CD8 T cells of HLA-A^*^0201 donors were used, and dose-dependent production of IFN-γ was also observed with this peptide (Figure 1C). We could not observe any considerable IFN-γ response with whole NY-ESO-1 protein in any of the TCRs and corresponding HLA types tested (Figures 1A–C).

To test the capacity of the manufactured nanoparticles to elicit an immune response, nanoparticles containing whole NY-ESO-1 protein or NY-ESO-1 derived peptides were loaded onto moDCs and cultured with TCR transfected T cells. T cells stimulated with moDCs loaded with nanoparticles containing whole NY-ESO-1 protein produced low levels of IFN-γ even when high nanoparticle concentrations were used, and independent of the TCRs (Figure 1D, Supplementary Figure 1A). In contrast, when moDCs were loaded with nanoparticles containing either HLA-A2.1 binding short peptide-3 (157–165) or long peptide-3-LP (153–167) or HLA-DRB1 binding peptide-2 (117–143), high amounts (20–160-fold for the NY-ESO-1_157−165_ epitope) of IFN-γ were observed (Figure 1D). These results demonstrate that peptides derived from NY-ESO-1 are functional and more preferable for encapsulation within PLGA nanoparticles than the complete recombinant NY-ESO-1 protein.

### All Three NY-ESO-1 Peptides and IMM60 Are Functional Within Particles

PLGA nanoparticles produced with different amounts of peptides added per 1 mg PLGA were analyzed for peptide content. As expected, peptide content was higher in particles produced in the presence of high amounts of peptides. The amount of peptide that could be added during the production process was limited by its solubility in DMSO (Supplementary Figure 2, Supplementary Table 3).

**Figure 2:**
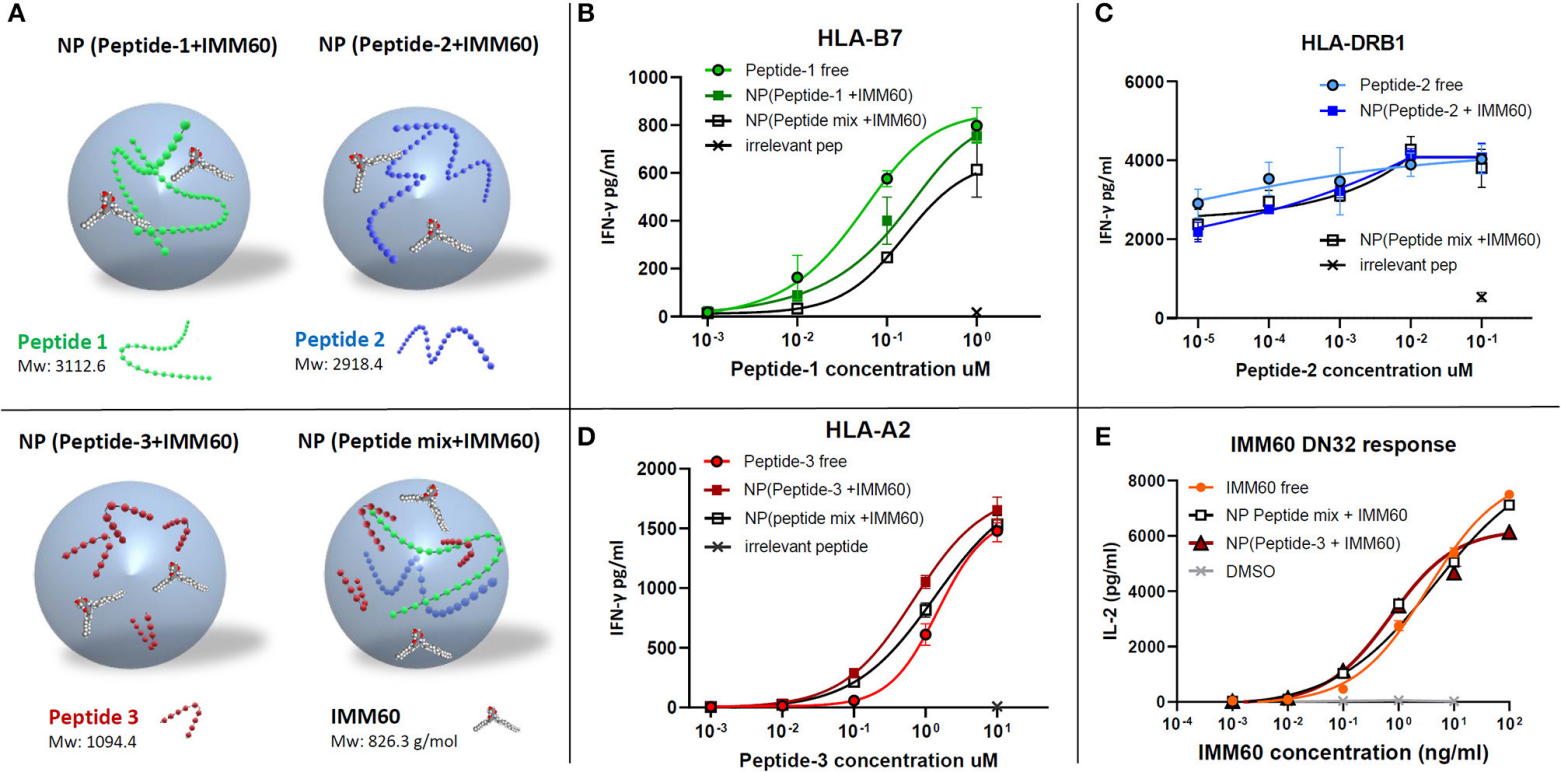
**(A)** Schematic demonstration of nanoparticles produced with different components. **(B–D)** Dose/IFN-γ response curves of free (dissolved in DMSO) or nanoparticle (NP) encapsulated peptides obtained by TCR transfected T cell cultures *in vitro*. **(B)** CD8+ T cell response against peptide-1 presented by HLA-B7, **(C)** CD4+ T cell response against peptide-2 presented by HLA-DRB1, and **(D)** CD8+ T cell response against peptide-3 presented by HLA-A2. Free peptides in solution (circles), nanoparticles containing only one peptide (colored squares), a mixture of three peptides (empty squares), and an irrelevant peptide at the highest concentration (cross) were shown. **(E)** Dose-dependent IL-2 production by DN32 mouse NKT cell hybridoma activated by IMM60 *in vitro*. Free IMM60 in solution (circles), nanoparticles containing all three peptides and IMM60 (1.39 ug/mg) (empty squares), nanoparticles containing only peptide-3 and IMM60 (1.42 ug/mg) (colored triangles) and DMSO (cross) were shown. Each dot represents the mean value of triplicate wells. Assays are performed with three different nanoparticle batches and figures are representative of four experiments with similar results. Response curves are fitted by four or five parameter non-linear regression analyses. Mean values are shown with SD.

Nanoparticles are primarily taken up by professional APCs such as DCs and macrophages and are useful carriers to deliver water-insoluble compounds or peptide/protein cargo which are prone to extracellular degradation (28, 29). Encapsulation of peptides may result in a poor release, degradation, or inability to escape to the cytosol once taken up by APCs, hampering cross-presentation. Therefore, antigen presentation of the nanoparticle-encapsulated peptides was compared to the free peptides (dissolved in DMSO). An overview of the different nanoparticles containing NY-ESO-1 peptides used in the study is given in Figure 2A. The IFN-γ production by TCR-transfected CD8 T cells was slightly less with the nanoparticle encapsulated peptide-1 (85–111) compared with free peptide-1 (Figure 2B). CD4 T cell response toward peptide-2 (117–143), already observed at a very low dose of the peptide, was not affected by the encapsulation procedure (Figure 2C). Further dilutions were performed with shorter incubation times or without LPS stimulation to reduce the response to this peptide. However, these changes also gave rise to higher inter and intra-assay variations in low doses with multiple nanoparticle batches and the encapsulated peptides performed at least equal when compared to soluble peptides (Supplementary Figures 2C,D). CD8 T cell response against peptide-3 (157–165) was also not affected (Figure 2D). Moreover, encapsulating all three peptides together neither affected the amount of each peptide within the nanoparticle (Supplementary Table 3B, Supplementary Figure 2B) nor their immunogenicity compared to the single encapsulated peptides (Figures 2B–D).

The functionality of the iNKT cell agonist, IMM60, in PLGA nanoparticles was analyzed by comparing the ability of free IMM60 or encapsulated IMM60 to induce IL-2 production by the mouse iNKT cell hybridoma DN32. Mouse JAWS-II DCs were loaded with IMM60-containing particles or free IMM60. No difference in IL-2 production by the mouse iNKT cells was observed between the two forms of IMM60, indicating that the nanomanufacturing process did not influence the functionality of IMM60 (Figure 2E).

In summary, all three selected NY-ESO-1 peptides and IMM60 are processed and presented by DCs to T cells when delivered together in PLGA nanoparticles.

### Patient-Derived T Cells Are Stimulated by Encapsulated Peptide-Loaded Autologous B Cells

**Figure 3:**
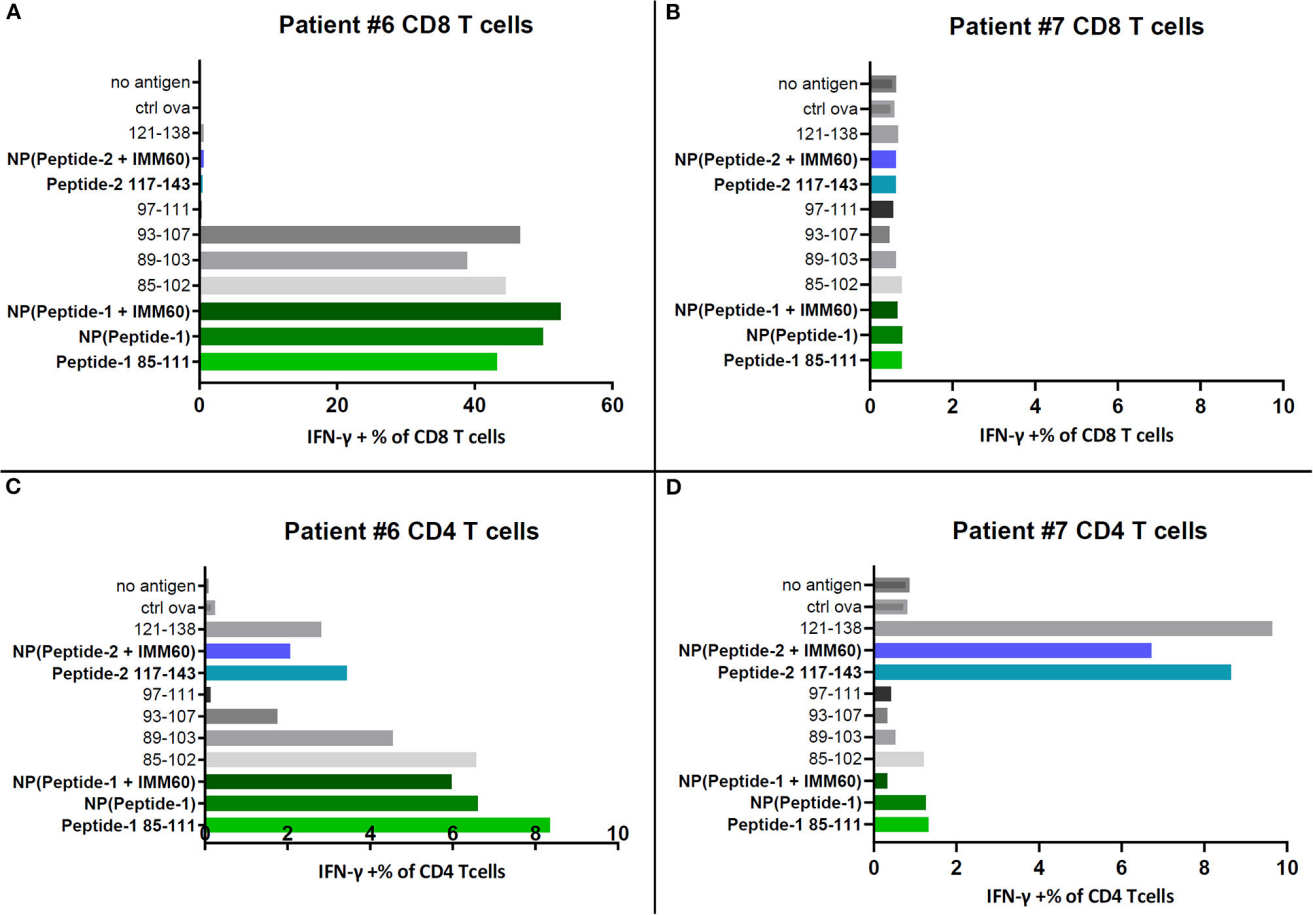
IFN-γ production by PBMC samples of two patients restimulated with NY-ESO-1 derived overlapping peptides in solution or selected free peptides (dissolved in DMSO) or nanoparticles. **(A)** IFN-γ positive CD8 T cells in restimulated PBMCs of patient#6, **(B)** IFN-γ positive CD8 T cells in restimulated PBMCs of patient#7, **(C)** IFN-γ positive CD4 T cells in restimulated PBMCs of patient#6, **(D)** IFN-γ positive CD4 T cells in restimulated PBMCs of patient#7.

To test a broader response, including other epitopes covered by the selected peptides, T cells within the PBMC of two patients (#6 and #7) that participated in a NY-ESO-1 vaccination trial were used (20). Autologous EBV-transformed B cells loaded with free peptides or peptide-containing PLGA nanoparticles were used as APCs. Encapsulated peptide-1 was recognized by CD8 T cells of patient #6 (Figure 3A). This patient has the HLA-B35 allele, which is known to present NY-ESO-1 epitopes 92–100 and 94–104 (Table 1, Supplementary Tables 4A,B). Indeed, CD8 T cell responses against soluble peptides 85–102, 89–103, and 93–107 covering the same epitopes were also observed (Figure 3A) (30). No CD8 T cell responses were detected in the PBMC of patient #7 which might be explained by the lack of MHC class I alleles known to present a NY-ESO-1 epitope (Figure 3B, Supplementary Table 4B). CD4 T cell responses were observed in both patients. Patient #6 had CD4 T cell responses against peptide-1 and peptide-2 (Figure 3C); the latter may correspond to a DRB1^*^0101 epitope covered by peptide-2 (Table 1). Furthermore, CD4 T cell responses against peptide-2 were also detected in patient #7, possibly presented by DRB1^*^0401 (Figure 3D, Supplementary Tables 4A,B). This experiment was repeated by directly introducing the antigens onto the PBMC cultures and without preloading onto autologous B cells. The trends in response to peptides were identical (Supplementary Figure 3A). CD8 T cell responses were heavily influenced by the lack of autologous B cells as antigen presenting cells which can be seen by the lower values of IFN-gamma producing cells in the latter setting (Supplementary Figure 3A). In comparison, CD4 T cell activation was less dependent on the B cell presentation.

Peptide-3 can only bind to HLA-A2.1; therefore, a broader response is not expected. The response to peptide-3 was tested with a patient-derived T-cell clone (4D8) cultured with EBV-transformed B cells as APCs. Similar stimulation of T cells was observed with a slightly higher activity of nanoparticle-encapsulated peptide-3 over its soluble counterpart in lower amounts (Supplementary Figure 3B).

### Co-encapsulation of Peptides and IMM60 Enhances CD4 T Cell Responses *in vivo*

**Figure 4:**
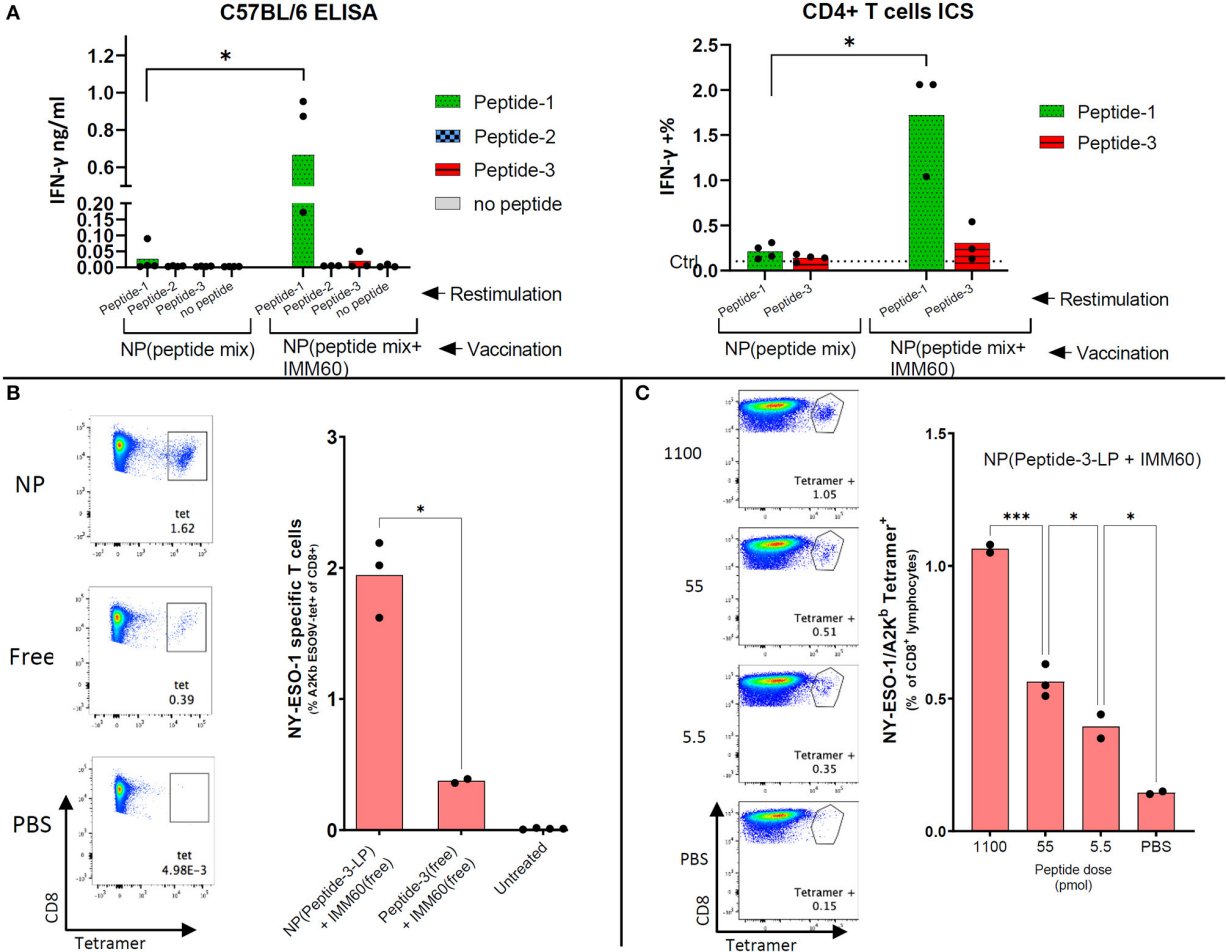
Endogenous CD4 and CD8 responses obtained by nanoparticle vaccination of mice. **(A)** C57Bl/6 mice were intravenously injected with 1 mg nanoparticles (NP) containing all three peptides with or without IMM60. Left: 7 days later, splenocytes were isolated and restimulated separately with peptide-1, peptide-2, or peptide-3. IFN-γ amounts in culture supernatants were determined 48 h later. Right: Splenocytes were restimulated *ex-vivo* either with peptide-1 or peptide-3 and stained with CD3, CD4, and intracellularly IFN-y. IFN-y staining in non-vaccinated mouse cells is shown as a dashed line. Dots represent each mouse. **(B)** F1 (HHD × C57BL/6J) mice were intravenously injected with 1.1 ug (0.6 nmol) peptide-3-LP either in solution or encapsulated within nanoparticles mixed with 50 ng IMM60 in solution. Twenty eight days later all mice were boosted with 1.1 ug peptide-3-LP in a solution mixed with 50 ng IMM60 in solution. Eight days after boost splenocytes were isolated and stained with HLA-A2/K^b^ NY-ESO-1_157−165_ tetramers. FACS plots show the NY-ESO-1_157−165_ specific CD8 T cells (in the square). Unpaired *t*-test with Welch's correction was used to determine statistical significance. **(C)** F1 (HHD × B6SJLCD45.1) mice were injected with nanoparticles containing Peptide-3-LP and IMM60 intravenously with different doses [2 ug (1.1 nmol), 100 ng (55 pmol), or 10 ng (5.5 pmol)] and 10 days later, splenocytes were isolated and stained with HLA-A2/K^b^ NY-ESO-1_157−165_ tetramers. Mean values are shown. Dots represent each mouse. One-way ANOVA with Tukey's correction was used to determine statistical significance.

Endogenous CD4 T cell responses in wild type C57BL/6 mice are known to generate a CD4 T cell response against NY-ESO-1 86–99 epitope (31). This sequence is also present in peptide-1. To test the induction of T cell responses *in vivo*, wild type C57BL/6 mice were injected with nanoparticles containing the three selected peptides either with or without IMM60. One week after a single injection, splenocytes were restimulated with the individual peptides. As expected, IFN-y production was only observed against peptide-1 (Figure 4A). Strikingly, IFN-y production by CD4 T cells was predominantly observed in mice injected with particles containing IMM60, suggesting that iNKT cells can provide help for CD4 T cell responses (Figure 4A). In conclusion, IMM60 enhanced CD4 T cell responses against NY-ESO-1 in wild type mice vaccinated with PLGA nanoparticles containing the selected NY-ESO-1 peptides and IMM60.

### Co-encapsulation of Peptides and IMM60 Enhances CD8 T Cell Responses *in vivo*

We could not observe any specific CD8 T cell response in wild type C57BL/6 mice (Supplementary Figure 3C). Therefore, we used both AAD and HHD mice which express a hybrid MHC class I molecule with α1 and α2 domains of the human HLA-A2.1 molecule fused to α3 domain of mouse H-2D^b^ molecule. In HHD mice, HLA-A2.1 heavy chain was covalently linked to the human β2 m light chain, denominated HHD molecule, and the H-2 Db and mouse β2-microglobulin genes have been disrupted preventing the expression of MHC class I K^b^ (as well as Class I like molecules such as CD1d) (32). In the F1 (HHD × C57BL/6J) generation of HHD mice, wild-type murine β2-microglobulin is expressed, allowing for the expression of CD1d, and thus the normal development of iNKT cells.

For experiments in HLA-A2 transgenic mice, we initially used the longer version of peptide-3 [peptide-3-LP (153–167)] presented by HLA-A2. This peptide induced lower IFN-γ responses than peptide-3 (157–165) in equimolar concentrations *in vitro* and both in free and nanoparticle encapsulated forms (Supplementary Figure 4A). To test the activity of the nanoparticles containing peptide-3-LP *in vivo*, adoptively transferred NY-ESO-1_157−165_ specific mouse CD8 T cells (m1G4) were tested for their capacity to respond. CD8 T cells were fluorescently labeled and transferred to F1 (C57BL/6 × HHD) mice which were vaccinated with nanoparticles the next day. Nanoparticles containing peptide-3-LP were able to stimulate adoptively transferred CD8 T cells albeit with low sensitivity (2 nmol of the long peptide stimulated m1G4 T cell, 0.1 nmol failed to do so) (Supplementary Figure 4B).

For the induction of NY-ESO-1_157−165_ specific T cells from the endogenous T cell repertoire, F1 (C57BL/6 × HHD) mice were injected intravenously with nanoparticles containing both peptide-3-LP as well as IMM60 or with soluble peptide-3-LP and soluble IMM60. After 28 days, a booster injection was given. Enhanced nanoparticle-mediated delivery of antigen was observed (Figure 4B). When nanoparticles containing peptide-3-LP and IMM60 were used, endogenous T cell responses could be detected even after a single dose up to 5.5 pmol 10 days after vaccination (Figure 4C). As a result, PLGA nanoparticles encapsulating NY-ESO-1 peptide-3-LP and IMM60 were able to expand endogenous antigen-specific CD8 T cells recognizing the HLA-A2 epitope.

**Figure 5:**
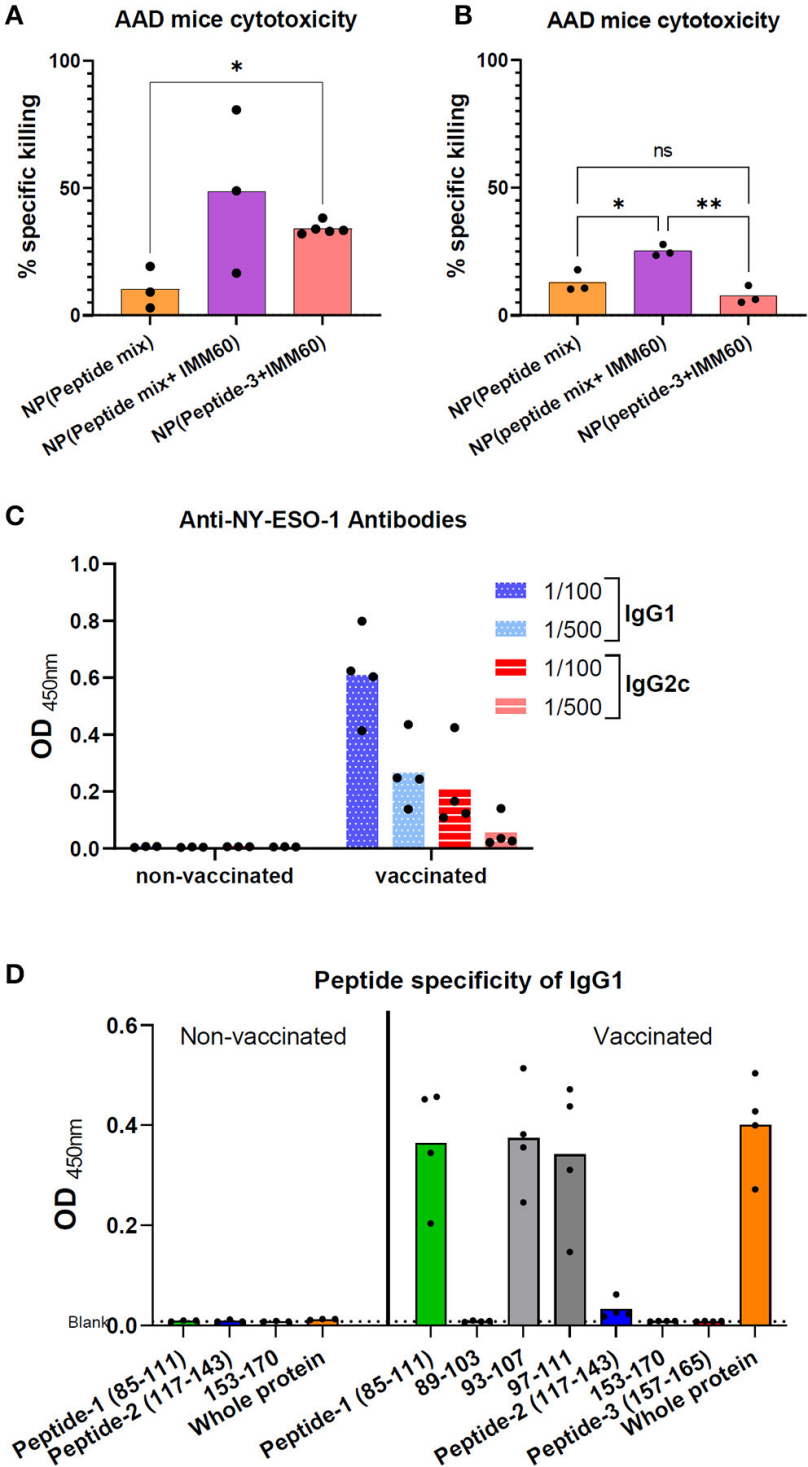
**(A)** Homozygous AAD mice were intravenously injected with nanoparticles encapsulating 6 ug (5.5 nmol) peptide-3 either mixed with peptide-1 and 2 [NP (Peptide mix)], mixed with peptide-1,2 and IMM60 [NP (peptide mix+IMM60)], or only with IMM60. All mice were boosted with the same nanoparticles on day 28. Seven days after booster injections, specific *in vivo* cytotoxicity of peptide-3 loaded target cells was analyzed. Dots represent each mouse, 3–5 mice were used per group. **(B)** Experiment in A is repeated without any booster injections and peptide-3 loaded target cells were transferred 7 days after a single injection of nanoparticles with a dose of each containing 6 ug peptide-3. Dots represent each mouse, 3 mice were used per group. **(C)** Anti-NY-ESO-1 antibody levels in the sera of AAD mice vaccinated with NP (peptide mix+IMM60) or non-vaccinated and inoculated with 1C12 sarcoma cells expressing full-length NY-ESO-1. Serum samples were diluted 1/100 and 1/500, optical densities of duplicate wells are shown. Dots represent each mouse. **(D)** Peptide specificity of anti-NY-ESO-1 antibodies in the sera of the same AAD mice as shown in **(C)**. Optical density of ELISA wells indicating relative IgG1 antibody levels is shown. Bars show mean optical density values of all mice, dots represent each mouse.

To further elucidate the role of IMM60 in CD8 T cell responses, homozygous AAD mice were used. In these mice, β2-microglobulin is intact; therefore, they have a similar expression of H2-Dd and HLA-A2.1 as well as other class I-like molecules such as CD1d (33) and have functional iNKT cells (Supplementary Figures 5A,B). AAD mice were vaccinated with nanoparticles containing all three peptides (peptide-1, peptide-2, peptide-3) with or without IMM60 or only peptide-3 with IMM60. After 2 monthly injections, target cells were loaded with peptide-3, transferred to immunized mice, and antigen-specific cytotoxicity was measured. The mice injected with nanoparticles containing IMM60 demonstrated higher peptide-3 specific CD8 T cell cytotoxicity than in mice injected with nanoparticles without IMM60 but a mixture of peptides (Figure 5A). When we repeated this experiment without a booster injection, the cytotoxic response against peptide-3 with nanoparticles containing a peptide mixture but not IMMM60 did not show a major change. However, cytotoxic responses against particles containing both the single and multiple peptides with IMM60 were reduced compared to the booster regime. Nevertheless, nanoparticles with peptide mix (containing a mouse CD4 T cell epitope) and IMM60 was superior to both the nanoparticles without IMM60 and nanoparticles with a single CD8 T cell-specific peptide and IMM60 (Figure 5B).

Together, these results demonstrate that PLGA encapsulated long peptides have higher CD8 T cell immunogenicity *in vivo* than freely administered formulations which can be further enhanced by co-delivery of IMM60.

### PLGA Nanoparticles With Peptides and IMM60 Enhance Antibody Responses Against NY-ESO-1

AAD mice inoculated with full-length NY-ESO-1 expressing 1C12 sarcoma tumor cells rejected the tumors spontaneously, and T cell responses were observed in most mice (Supplementary Figures 5C,D). Sera of these mice were analyzed to test if the nanoparticles containing three NY-ESO-1 peptides and IMM60 can boost antibody responses against NY-ESO-1. Despite a similar tumor rejection in both groups, NY-ESO-1 specific antibodies in IgG1 and IgG2c isotypes were detected exclusively in the sera of mice injected with nanoparticles containing three peptides and IMM60 (Figure 5C). When the sera of these mice were subjected to an ELISA with peptide coated wells, almost all reactivity against whole NY-ESO-1 protein could be reproduced with Peptide-1 (Figure 5D, Supplementary Figure 6). The B cell epitope was also preserved within the shorter 93–107 and 97–111 peptides (Figure 5D, Supplementary Figure 6). Since both groups were subjected to two challenges with NY-ESO-1 expressing tumors, injection of nanoparticles, containing both iNKT cell agonist and NY-ESO-1 peptides, are likely to have a critical contribution to antibody responses against NY-ESO-1.

### PLGA Nanoparticles With Peptides and IMM60 Have Reversible Side Effects in Preclinical Toxicology Studies

**Figure 6:**
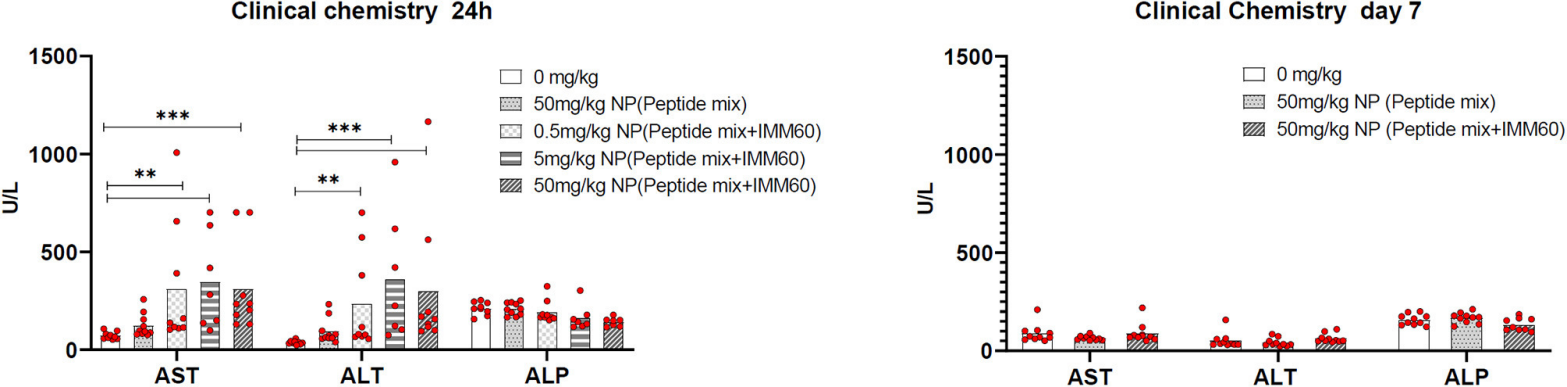
Wild type C57BL/6 mice were intravenously injected either with PBS (0 mg/kg), nanoparticles containing NY-ESO-1 peptide mix (6.1 ug Peptide-1, 4.3 ug Peptide-2, and 11 ug Peptide-3 per mg NP) (50 mg/kg NP peptide mix) or increasing doses of nanoparticles containing NY-ESO-1 peptide mix and IMM60 (8.8 ug Peptide-1, 5.9 ug Peptide-2 and 12 ug Peptide-3 with 1.2 ug IMM60 per mg NP) 0.5, 5, 50 mg/kg NP (peptide mix+IMM60). Total IMM60 doses for a mouse weighing 20 g were 0.012, 0.12, and 1.2 ug, respectively. Twenty-four hour and 7 days after injections Alanine aminotransferase (ALT), Aspartate aminotransferase (AST), and Alkaline phosphatase (ALP) levels were measured in blood plasma. Five male and 5 female, 10 mice were used for each group, dots represent each mouse, Kruskal-Wallis and Dunn's pairwise comparisons were performed for statistical analysis. Only significant differences were shown.

The toxicity of PLGA nanoparticles containing the 3 selected NY-ESO-1 peptides and IMM60 was tested in a preclinical toxicology study. During the toxicology study, no unscheduled deaths were observed. One day after injection, an increase in the liver enzymes AST and ALT was observed, which normalized at day 7, indicating transient liver toxicity (Figure 6). Moreover, necropsy results showed IMM60-associated liver toxicity [i.e., hepatocellular necrosis, (peri)vascular mononuclear cell infiltration, and thrombus formation] on day 1 without a dose-response relation (Supplementary Table 5). Also, minimal/mild pulmonary vascular mononuclear cell infiltration was observed.

On day 7, despite a low-grade granulomatous inflammation in one mouse and minimal (peri)vascular mononuclear cell infiltration in three mice, hepatocellular necrosis in the 50 mg/kg subgroup was no longer recorded (Supplementary Table 6), and thrombus formation was almost completely recovered. Similarly, the pulmonary findings observed in 3 out of 10 mice were recovered (Supplementary Table 6). In mice injected with nanoparticles encapsulating the 3 peptides only, hepatocellular apoptosis/necrosis was also observed. The lesions were, however, smaller and without thrombosis or mononuclear cell infiltration compared to the mice injected with nanoparticles containing IMM60.

In brief, the presence of minimal/mild thrombosis and necrosis in the liver 1 day after nanoparticle injection was considered to be adverse, while partial recovery of necrosis and almost complete recovery of thrombosis were noted within 7 days.

## Discussion

In our previous studies, iNKT cell agonists outperformed TLR ligands upon encapsulation in PLGA nanoparticles for the co-delivery of antigen and adjuvant to DCs (14). Effects were mediated by chemokines, CCL17, and CXCL9, induced by iNKT cells during the cross-priming of CD8 T cells (16). Besides, intravenous injection of nanoparticles was necessary for a robust anti-tumor immune response which can synergize with checkpoint modulation (15). However, so far, mostly ovalbumin protein was used in these studies as a model antigen. Here, we took the next step toward clinical application by moving to the widely expressed tumor-associated cancer germline antigen NY-ESO-1.

NY-ESO-1 can be qualified as the most suitable TAA for an “off the shelf” cancer vaccine due to its leading immunogenicity which has been reaffirmed by a recent mRNA vaccination trial incorporating various tumor-associated antigens (13). Although mRNAs encoding multiple full-length antigens can be easily encapsulated within liposomes, peptide/protein antigens within PLGA have the advantage of long-term stability and wide storage conditions. NY-ESO-1 whole protein is known to be poorly cross-presented when delivered to DCs in free form (19). The cross-presentation of NY-ESO-1 is enhanced by different formulations which were previously demonstrated to induce specific T cell responses in clinical trials (19, 20, 34). We also reported high T cell stimulation with nanoparticles made of the same full-length NY-ESO-1 and LPS coated with polyphenol (35). However, in this study, no considerable response with PLGA nanoparticles encapsulating NY-ESO-1 protein was observed. This could be due to the low solubility of the NY-ESO-1 protein in aqueous and organic solvents which hampers encapsulation and quantification. In contrast, peptides derived from NY-ESO-1 were shown to be effective for vaccination, and higher molar quantities of peptides could be encapsulated improving the immunogenicity of the PLGA nanoparticle vaccine (11, 36–38). Accordingly, we focused on immunogenic epitopes of NY-ESO-1 and determined two NY-ESO-1 long (85–111 and 117–143) peptides and one short (157–165) peptide which are presented in a range of MHC class-I and MHC class-II molecules covering more than 80% of the European population.

In a previous study, similar NY-ESO-1-derived peptides and α-GalCer loaded onto monocyte-derived DCs induced iNKT cell expansion and CD4 T cell responses in the majority, and CD8 T cell responses in some patients (38). Besides, we also added a third short peptide (peptide-3, 157–165) presented by the highly prevalent HLA-A2.1 molecule, which may improve CD8 T cell response rates. The same epitope was used previously in clinical trials but suffered from either a cryptic epitope emerging in longer forms due to extracellular cleavage or being presented by non-APCs in the short form (24, 39). Nanoparticle delivery can protect peptides from extracellular cleavage until professional APCs take them up, and spleen resident cDC1s can enhance priming of CD8 T cells by utilizing the CCL17 and CXCL9 networks (16, 40, 41). Indeed, we observed higher endogenous CD8 T cell responses against this epitope *in vivo* with PLGA nanoparticles, and short peptides could also induce cytotoxic T cell response in nanoparticle form. As expected, CD8 T cell cytotoxicity against NY-ESO-1_157−165_ is further enhanced by the presence of IMM60 within the particles reaffirming our previous findings on CD8 T cell responses against ovalbumin protein and HPV-E7 peptide (14, 15). Moreover, we observed even higher cytotoxic T cell responses when a CD4 T cell epitope is also introduced within particles together with IMM60. This hints at a further enhancement of iNKT cell help by CD4 T cell help when multiple epitopes are covered within the particles. Therefore, nanoparticle-mediated peptide delivery is expected to enhance CD8 T cell responses against 157–165 epitope together with the extended activation of human iNKT cells by the novel iNKT cell agonist IMM60 (14, 18).

Harnessing the helper functions of iNKT cells is also known to enhance antigen-specific CD4 T cell responses (42, 43). This was also relevant in our vaccination strategy with nanoparticles containing NY-ESO-1 peptides in which the presence of IMM60 primed endogenous CD4 T cells that were otherwise almost absent with particles containing only peptides. Tumor cells could also present MHC class-II epitopes of NY-ESO-1 on their surface, and NY-ESO-1 specific CD4 T cells were previously demonstrated to induce tumor regression in melanoma patients (44, 45). CD4 T cells can play various critical roles in sustaining an anti-tumor microenvironment, helping NK and CD8 T cell survival and cytotoxicity as well as B cell responses.

IC12 sarcoma line was transduced with a lentiviral vector to express full-length NY-ESO-1 and was cloned based on the highest expression of NY-ESO-1. Unfortunately, this also increased the immunogenicity of the tumor to cause spontaneous rejections with a visible CD8 T cell response hampering the detection of vaccine-induced T cell immunity. On the other hand, we could demonstrate a *de novo* antibody response against NY-ESO-1 protein in the IC12 sarcoma inoculated mice which were also vaccinated with nanoparticles containing three peptides and IMM60. Even though all mice have been introduced with NY-ESO-1 *via* the sarcoma cell line twice, the full-length antigen may not be easily accessible to the B cells as well as the nanoparticle delivered peptides or IMM60 could be responsible for transactivating the B cells in the vaccination group. Considering the CD4 T cell responses which were dependent on co-encapsulation of IMM60 but not the peptides, IMM60 is likely to have a significant contribution also to the antibody responses. INKT cells are known to provide both cognate and non-cognate help to B cells leading to enhanced antibody responses. Through cognate help, marginal zone B cells can acquire the particles and present glycolipid to iNKT cells. By contrast, by non-cognate help, DCs licensed by iNKT cells activate the CD4 helper T cells, which facilitates B cell stimulation (46, 47). However, further research is required to fully decipher the mechanism of help toward B cells in the PLGA nanoparticle-mediated peptide and agonist co-delivery settings.

As a final step toward clinical application, the systemic toxicity of PLGA nanoparticles containing three NY-ESO-1 peptides and IMM60 was evaluated. Mild and mostly transient liver toxicity was observed in mice treated with IMM60 containing nanoparticles indicating that the toxicity is dependent on iNKT cell activation, which is known to reside in the liver of mice in high numbers. Considering the lower numbers of iNKT cells in humans compared with mice, and the safety of the iNKT cell agonist α-GalCer in previous clinical treatments, the PLGA nanoparticle vaccines containing iNKT cell agonists can be regarded as a safe and effective modality. We believe that the long-term stability and a full spectrum of immunity could also render these nanoparticles with potential use against viral infections.

In summary, we demonstrated the feasibility to encapsulate three NY-ESO-1-derived peptides together with IMM60 in PLGA nanoparticles. All peptides are efficiently processed and presented by multiple HLA types *in vitro* and *in vivo* while preserving the potency of IMM60. Furthermore, iNKT cell help was provided for multiple epitope-specific CD8 and CD4 T cell responses, and a *de novo* antibody response was observed in NY-ESO-1 positive tumor-bearing mice *via* PLGA nanoparticle-mediated co-delivery. Finally, no serious adverse events occurred in the toxicological evaluation warranting the clinical testing of these nanoparticles.

## Data Availability Statement

The raw data supporting the conclusions of this article will be made available by the authors, without undue reservation.

## Ethics Statement

The animal study was reviewed and approved by Nijmegen Animal Experiments Committee (Project No: DEC2015-019 and DEC2019-020) and the Oxford Animal Welfare Ethics Review Board (Home Office under license number PBA43A2E4).

## Author Contributions

IdV, CF, and VC conceived the research question. YD, UG, and MV designed the peptides and experiments. EV produced all nanoparticles, analyzed peptide contents and physical characteristics, contributed to tissue processing, and ELISA's. JC assisted in designing the toxicology study and interpreting the results. NvR assisted in *in vivo* experiments. EJ and MH developed the method for the IMM60 quantification and performed IMM60 content analysis. MD supervised TCR transfection and antigen presentation assays and contributed to the interpretation of the results. YD, UG, and J-LC planned and performed the experiments, processed the experiment data, designed the figures, and interpreted results. VC and CF supervised the experiments. YD drafted. UG supplemented, reviewed, and edited. J-LC, MV, EJ, MH, MD, and JC edited. CF and IdV reviewed and edited. IdV revised the manuscript. All authors contributed to the article and approved the submitted version.

## Conflict of Interest

The authors declare that the research was conducted in the absence of any commercial or financial relationships that could be construed as a potential conflict of interest.
