# Supplementary material for: PLGA Nanoparticles Co-encapsulating NY-ESO-1 Peptides and IMM60 Induce Robust CD8 and CD4 T Cell and B Cell Responses
Source: Front Immunol. 2021 Feb 25;12:641703. doi: 10.3389/fimmu.2021.641703 (PMC7947615; doi:10.3389/fimmu.2021.641703)
Supplement: Supplementary file 1 [file Table_1.pdf]

**Supplementary Table-1** : HLA types reported to present an epitope within the relevant peptides are listed according to http://tools.iedb.org/population/. HLA allele frequency within all Europe without any specific country or ethnicity is written in paranthesis.

European Coverage Class-I HLA

| Epitope | Coverage | HLA allele (genotypic frequency (%)) | | | | | | | | | | | | Total HLA hits |
|---|---|---|---|---|---|---|---|---|---|---|---|---|---|---|
| | Class I | HLA-A*02:01 (28.14) | HLA-A*24:02 (9.42) | HLA-A*68:01 (3.34) | HLA-B*07:02 (11.91) | HLA-B*35:01 (5.65) | HLA-B*35:08 (0.48) | HLA-B*49:01 (1.36) | HLA-B*51:01 (5.21) | HLA-B*52:01 (1.33) | HLA-C*03:03 (4.75) | HLA-C*03:04 (6.01) | HLA-C*12:02 (1.32) | |
| Peptide-1 SRLLEFYLAMPFATPMEAELARRSLAQ | 64.49% | - | + | + | + | + | + | - | + | + | + | + | + | 10 |
| Peptide-2: PVPGVLLKEFTVSGNILTIRLTAADHR | 8.77% | - | - | + | - | - | - | + | - | - | - | - | - | 2 |
| Peptide-3: SLLMWITQC | 47.00% | + | - | - | - | - | - | - | - | - | - | - | - | 1 |
| Epitope set | 83.68% | 1 | 1 | 2 | 1 | 1 | 1 | 1 | 1 | 1 | 1 | 1 | 1 | 13 |

European Coverage Class-II HLA

| **Epitope** | **Coverage** | **HLA allele (genotypic frequency (%))** | | | | | | | | | **Total HLA hits** |
|---|---|---|---|---|---|---|---|---|---|---|---|
| | **Class II** | **HLA-DPB1*04:01 (38.47)** | **HLA-DPB1*04:02 (10.89)** | **HLA-DQB1*04:01 (1.05)** | **HLA-DRB1*01:01 (8.22)** | **HLA-DRB1*04:01 (6.20)** | **HLA-DRB1*07:01 (13.08)** | **HLA-DRB1*09:01 (1.27)** | **HLA-DRB1*11:01 (6.78)** | **HLA-DRB1*16:01 (4.12)** | |
| **Peptide -1: SRLLEFYLAMPFATPMEAELARRSLAQ** | **84.45%** | + | + | + | + | + | + | + | - | - | **7** |
| **Peptide-2: PVPGVLLKEFTVSGNILTIRLTAADHR** | **42.78%** | - | - | - | + | + | - | - | + | + | **4** |
| **Peptide-3: SLLMWITQC** | **69.64%** | - | - | - | - | - | - | - | - | - | **2** |
| **Epitope set** | **88.63%** | **1** | **1** | **1** | **2** | **2** | **1** | **1** | **1** | **1** | **11** |

- : not restricted

**Supplementary Table-2: A)**TCRs coded by mRNA's used in in vitro stimulation assays. Known recognizing minimal peptides, and binding HLA types. **B)** Physical characteristics of each peptide.

A)

| TCR name | Specific peptide | position | HLA type |
|---|---|---|---|
| CD8-pST1-hTCR-NYE#12 | ATPMEAELARRSLAQ | 97-111 | B*0702 |
| CD4-pST1-hTCR-NYE#3 | PVPGVLLKEFTVSGNILTIRLT | 117–139 | DRB1*0401 |
| CD8-pST1-NY-ESO 1-A2-TCR-VhuCmu | SLLMWITQC | 157-165 | A*0201 |

B)

| Name | Position | Sequence | Hydropathy<br>Top is hydrophilic<br>Bottom is hydrophobic | Iso-electric point<br>solubility |
|---|---|---|---|---|
| **Peptide 1** | 85-111 (27mer) | **SRLLEFYLAMPFATPMEAELARRSLAQ** | 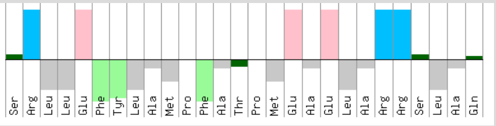 | pH 6.82<br>≤5mg/ml in $H_2O$<br>≤10mg/ml in DMSO |
| **Peptide 2** | 117–143 (27mer) | **PVPGVLLKEFTVSGNILTIRLTAADHR** | 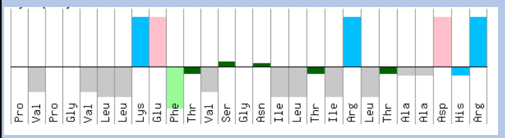 | pH 10.52<br>≤10mg/ml in $H_2O$<br>≤5mg/ml in DMSO |
| **Peptide 3** | 157-165 (9mer) | **SLLMWITQC** | 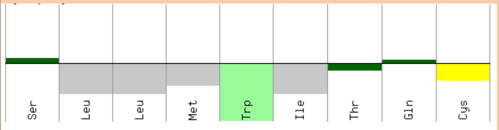 | pH 3.00<br>Undissolved in $H_2O$<br>≤10mg/ml in DMSO |

Color codes: Acidic Aromatic Basic Aliphatic Polar Cysteine

**Supplementary Table-3: A)** Nanoparticles containing single peptides. **B)** Nanoparticles containing three peptide mix. Peptides used in production, peptide content after production and washing, and size characteristics of each nanoparticle batch are mentioned in different rows.

A)

| Encapsulation of single peptides by PLGA | | | | |
|---|---|---|---|---|
| Peptide | ug peptide used per mg PLGA | Peptide content of nanoparticle (ug pep/mg NP) | Particle Size ± SD | IMM60 |
| Peptide 1 | 5 | 4.2 | 177±50 | + |
| | 5 | 1.3 | 211±70 | + |
| | 10 | 3.1 | 178±47 | + |
| | 15 | 4.6 | 215±66 | + |
| | 22.5 | 12.8 | 213±51 | + |
| | 22.5 | 12.4 | 208±49 | - |
| Peptide 2 | 5 | 1 | 184±54 | + |
| | 10 | 9.7 | 203±53 | + |
| | 20 | 22.4 | 224±71 | + |
| Peptide 3 | 5 | 5.8 | 198±52 | + |
| | 10 | 7.1 | 201±55 | + |
| | 20 | 11.3 | 218±59 | + |

B)

| Encapsulation of multiple peptides by PLGA | | | | | |
|---|---|---|---|---|---|
| ug each peptide used per mg PLGA | Peptide content of nanoparticle (ug pep/mg NP) | | | Particle Size | IMM60 |
| | Peptide 1 | Peptide 2 | Peptide 3 | | |
| 5 | 6.5 | 4.3 | 6.8 | 189±54 | + |
| 5 | 7.1 | 4 | 4 | 203±58 | + |
| 10 | 8.2 | 7.2 | 8.3 | 211±66 | + |
| 10 | 7.7 | 6.4 | 9.5 | 212±73 | + |
| 10 | 7.2 | 6.2 | 9.3 | 219±60 | + |
| 10 | 11.4 | 13.4 | 11.5 | 227±61 | + |
| 10 | 7.6 | 7.1 | 7.6 | | |
| 10 | 6.2 | 5.5 | 7.5 | | |
| 10 | 8.8 | 5.9 | 12 | 199±51 | + |
| 10 | 6.1 | 4.3 | 11 | 201±53 | - |

**Supplementary Table-4:A)** Known B35 binding epitope (underlined) within the peptide-1 and DRB1*0401 epitope (underlined) within peptide-2 shown with other overlapping peptides used in figure-3. **B)** HLA genotyping results of patients #6 and #7 in figure-3

A)

| **Peptide sequence** | **Position** | **Name** |
|---|---|---|
| SRLLEFYLAMPFATPMEAELARRSLAQ | 85-111 | Peptide-1 |
| SRLLEFYLAMPFATPMEA | 85-102 | |
| EFYLAMPFATPMEAE | 89-103 | |
| AMPFATPMEAELARR | 93-107 | |
| ATPMEAELARRSLAQ | 97-111 | |
| PVPGVLLKEFTVSGNILTIRLTAADHR | 117–143 | Peptide-2 |
| VLLKEFTVSGNILTIRLT | 121-138 | |

B)

Patient 06

| | | | | | | |
|---|---|---|---|---|---|---|
| **HLA Type - Class I** | A3 | A1 | **B35** | B8 | Cw4 | Cw7 |
| **HLA Type – Class II** | **DRB1*01** | DRB1*03 | DRB3*01 | DQB1*02 | DQB1*05 | |

Patient 07

| | | | | | | |
|---|---|---|---|---|---|---|
| **HLA Type - Class I** | A1 | A2 | B8 | B27 | Cw2 | Cw7 |
| **HLA Type – Class II** | DRB1*0301/11 | **DRB1*0401/** 13/16/21/26/ 33/35/38 | DRB3*01 | DRB4*01 | DQB1*0201 | DQB1*0302 |

**Supplementary Table-5**:PLGA NY-ESO-1 nanoparticle related microscopic findings in liver and lung – scheduled euthanasia animals (Day 1).

| | **Males** | | | | | **Females** | | | | |
|---|---|---|---|---|---|---|---|---|---|---|
| **Group** | **1** | **2** | **3** | **4** | **5** | **1** | **2** | **3** | **4** | **5** |
| **Dose (mg/kg/day)** | **0** | **50** | **0.5** | **5** | **50** | **0** | **50** | **0.5** | **50** | **50** |
| **No. animals examined** | **5** | **5** | **5** | **5** | **5** | **5** | **5** | **5** | **5** | **5** |
| **Liver(No. Examined)** | (5) | (5) | (5) | (5) | (5) | (5) | (5) | (5) | (5) | (5) |
| Apoptosis/necrosis; hepato- celular, focal/multifocal | 0 | 5 | 5 | 4 | 5 | 0 | 5 | 1 | 3 | 5 |
| Minimal | 0 | 0 | 3 | 2 | 3 | 0 | 2 | 1 | 0 | 3 |
| Mild | 0 | 5 | 2 | 2 | 2 | 0 | 3 | 0 | 1 | 1 |
| Moderate | 0 | 0 | 0 | 0 | 0 | 0 | 0 | 0 | 2 | 1 |
| Infiltration, mononuclear cell; vascular/perivascular | 0 | 0 | 4 | 5 | 4 | 0 | 0 | 5 | 5 | 5 |
| Minimal | 0 | 0 | 4 | 3 | 4 | 0 | 0 | 3 | 1 | 0 |
| Mild | 0 | 0 | 0 | 2 | 0 | 0 | 0 | 2 | 4 | 5 |
| Thrombus | 0 | 0 | 3 | 3 | 2 | 0 | 0 | 2 | 5 | 3 |
| Minimal | 0 | 0 | 1 | 0 | 2 | 0 | 0 | 1 | 2 | 1 |
| Mild | 0 | 0 | 2 | 3 | 0 | 0 | 0 | 1 | 3 | 1 |
| Moderate | 0 | 0 | 0 | 0 | 0 | 0 | 0 | 0 | 0 | 1 |
| **Lung (No. Examined)** | (5) | (5) | (5) | (5) | (5) | (5) | (5) | (5) | (5) | (5) |
| Infiltration, mononuclear cell; vascular | 0 | 0 | 5 | 5 | 3 | 0 | 0 | 4 | 5 | 5 |
| Minimal | 0 | 0 | 5 | 5 | 3 | 0 | 0 | 4 | 3 | 4 |
| Mild | 0 | 0 | 0 | 0 | 0 | 0 | 0 | 0 | 2 | 1 |

**Supplementary Table-6:** PLGA NY-ESO-1 nanoparticle related microscopic findings in liver and lung –Scheduled Euthanasia Animals (Day 7).

| | **Males** | | | **Females** | | |
|---|---|---|---|---|---|---|
| **Group** | **1** | **2** | **5** | **1** | **2** | **5** |
| **Dose (mg/kg)** | **0** | **50** | **50** | **0** | **50** | **50** |
| **No. animals examined** | **5** | **5** | **5** | **5** | **5** | **5** |
| **Liver (No. Examined)** | (5) | (5) | (5) | (5) | (5) | (5) |
| Apoptosis/necrosis; hepatocellular | 0 | 2 | 3 | 0 | 1 | 0 |
| Minimal | 0 | 2 | 2 | 0 | 1 | 0 |
| Mild | 0 | 0 | 1 | 0 | 0 | 0 |
| Infiltration, mononuclear cell; vascular/perivascular | 0 | 0 | 5 | 0 | 0 | 5 |
| Minimal | 0 | 0 | 5 | 0 | 0 | 2 |
| Mild | 0 | 0 | 0 | 0 | 0 | 3 |
| Granulomatous inflammation | 0 | 0 | 1 | 0 | 0 | 0 |
| Minimal | 0 | 0 | 0 | 0 | 0 | 0 |
| Mild | 0 | 0 | 1 | 0 | 0 | 0 |
| Thrombus, minimal | 0 | 0 | 1 | 0 | 0 | 0 |
| **Lung (No. Examined)** | (5) | (5) | (5) | (5) | (5) | (5) |
| Infiltration, mononuclear cell; vascular/perivascular | 0 | 0 | 1 | 0 | 0 | 2 |
| Minimal | 0 | 0 | 1 | 0 | 0 | 2 |
| Mild | 0 | 0 | 0 | 0 | 0 | 0 |
