# Supplementary material for: PLGA Nanoparticles Co-encapsulating NY-ESO-1 Peptides and IMM60 Induce Robust CD8 and CD4 T Cell and B Cell Responses
Source: Front Immunol. 2021 Feb 25;12:641703. doi: 10.3389/fimmu.2021.641703 (PMC7947615; doi:10.3389/fimmu.2021.641703)
Supplement: Supplementary file 2 [file Data_Sheet_1.pdf]

Supplementary Figure-1

A)

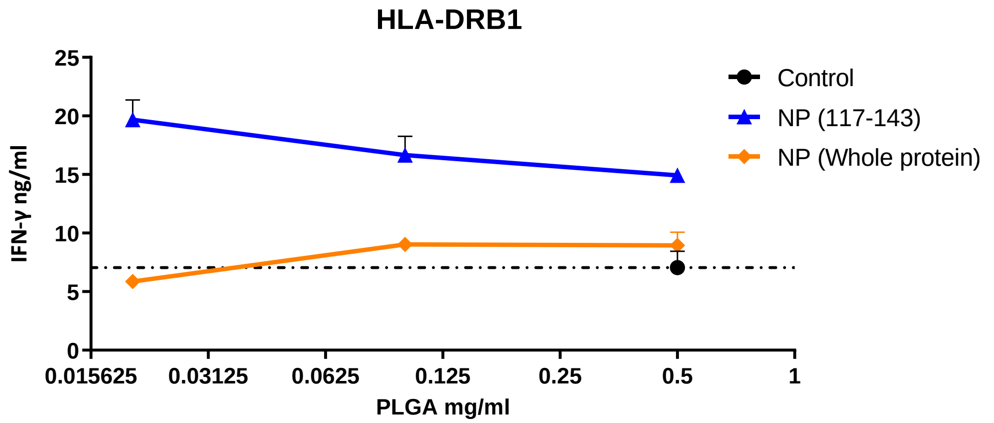

B)

GARGPESRLLEFYLAMPFATPMEAELARRS*(1,2)*
GARGPESRLLEFYLAMPFATPMEAELARRSLAQDAPPL*(3)*
SRLLEFYLAMPFATPMEAELARRSLAQ peptide-1
YLAMPFATPMEAELARRSLA*(4)*
MEAELARRSLAQDAPPLPVPGVLLKEFTVS*(1)*
PVPGVLLKEFTVSGNILTIRLTAADHR peptide-2
VPGVLLKEFTVSGNILTIRLTAADHR*(3)*
VLLKEFTVSGNILTIRLTAADHRQLQLSIS*(1)*

1.Wada H, Isobe M, Kakimi K, Mizote Y, Eikawa S, Sato E*, et al.* Vaccination with NY-ESO-1 overlapping peptides mixed with Picibanil OK-432 and montanide ISA-51 in patients with cancers expressing the NY-ESO-1 antigen. J Immunother 2014;37(2):84-92 doi 10.1097/CJI.0000000000000017.
2.Baumgaertner P, Costa Nunes C, Cachot A, Maby-El Hajjami H, Cagnon L, Braun M*, et al.* Vaccination of stage III/IV melanoma patients with long NY-ESO-1 peptide and CpG-B elicits robust CD8(+) and CD4(+) T-cell responses with multiple specificities including a novel DR7-restricted epitope. Oncoimmunology 2016;5(10):e1216290 doi 10.1080/2162402X.2016.1216290.
3.Gasser O, Sharples KJ, Barrow C, Williams GM, Bauer E, Wood CE*, et al.* A phase I vaccination study with dendritic cells loaded with NY-ESO-1 and alpha-galactosylceramide: induction of polyfunctional T cells in high-risk melanoma patients. Cancer Immunol Immunother 2018;67(2):285-98 doi 10.1007/s00262-017-2085-9.
4.Kakimi K, Isobe M, Uenaka A, Wada H, Sato E, Doki Y*, et al.* A phase I study of vaccination with NY-ESO-1f peptide mixed with Picibanil OK-432 and Montanide ISA-51 in patients with cancers expressing the NY-ESO-1 antigen. Int J Cancer 2011;129(12):2836-46 doi 10.1002/ijc.25955.

Supplementary Figure-1: A)CD4+ T cell response against nanoparticle encapsulated117-143 peptide presented by HLA-DRB1. Nanoparticles containing only peptide, and the whole NY-ESO-1 protein is compared by total PLGA nanoparticle concentration. **B)** The sequences of peptides previously used in vaccination studies in relation to the peptides used in this study. References are within the paranthesis.

Supplementary Figure 2

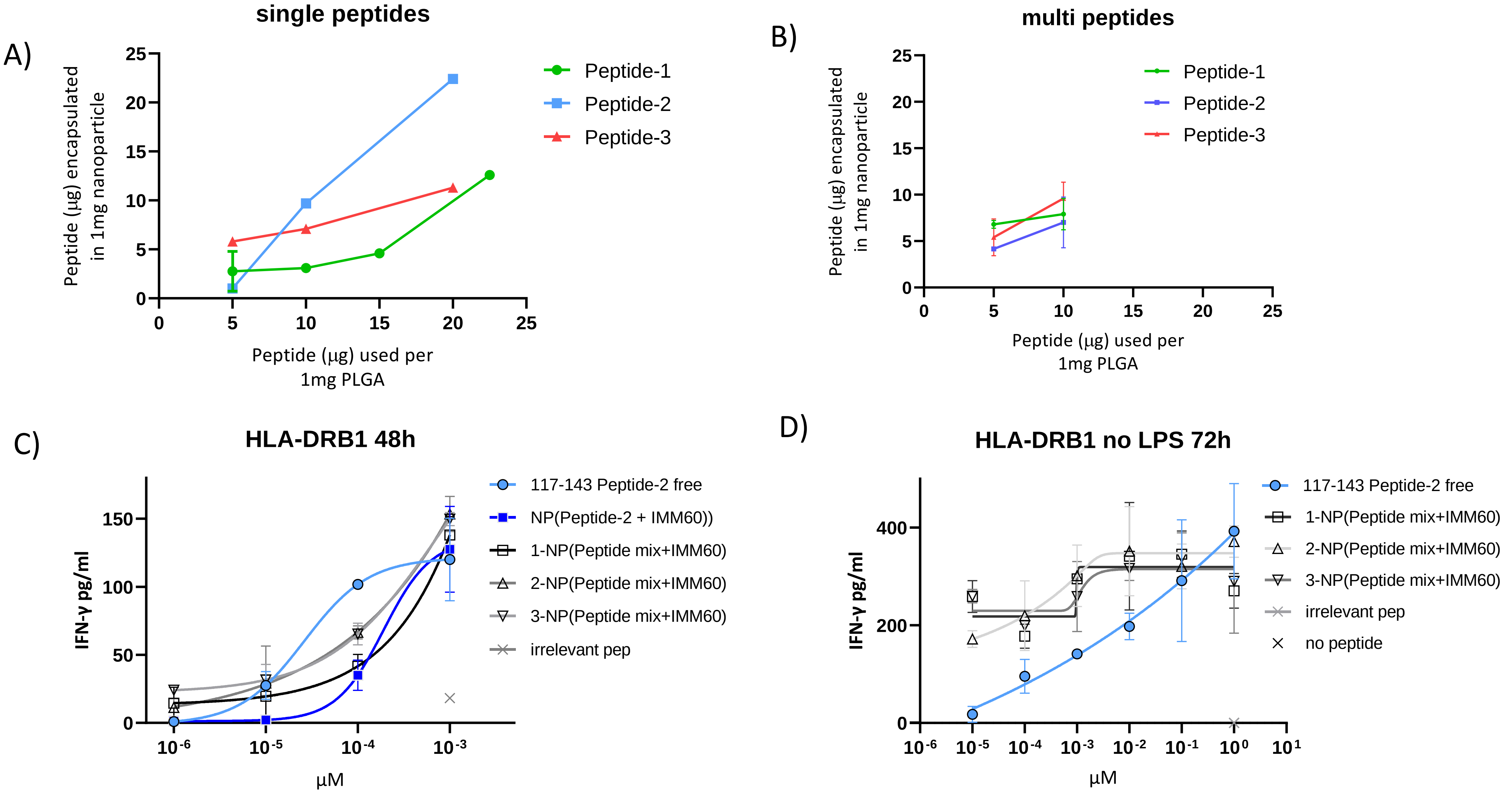

Supplementary figure-2: A) Encapsulation of single peptides within 1mg PLGA nanoparticle in relation to the amount of peptide used in production per 1mg PLGA. **B)** Encapsulation of each peptide within 1mg of PLGA nanoparticle in relation to the amount of each peptide within the peptide mix used in production per 1mg PLGA. **C-D)** CD4+ T cell response against peptide-2 presented by HLA-DRB1 as demonstrated in figure 2-C. **C**) Incubation time with TCR transfecte T cells were reduced to 48 hours**. D)** LPS was not added during the i72h incubation period with T cells. Free peptideds in solution (circles) , nanoparticles containing only one peptide (coloured squares), three different batches of particles containing a mixture of three peptides (empty squares or triangles), and an irrelevant peptide in highest concentration (cross) were shown. Mean values are shown with SD.

Supplementary Figure 3

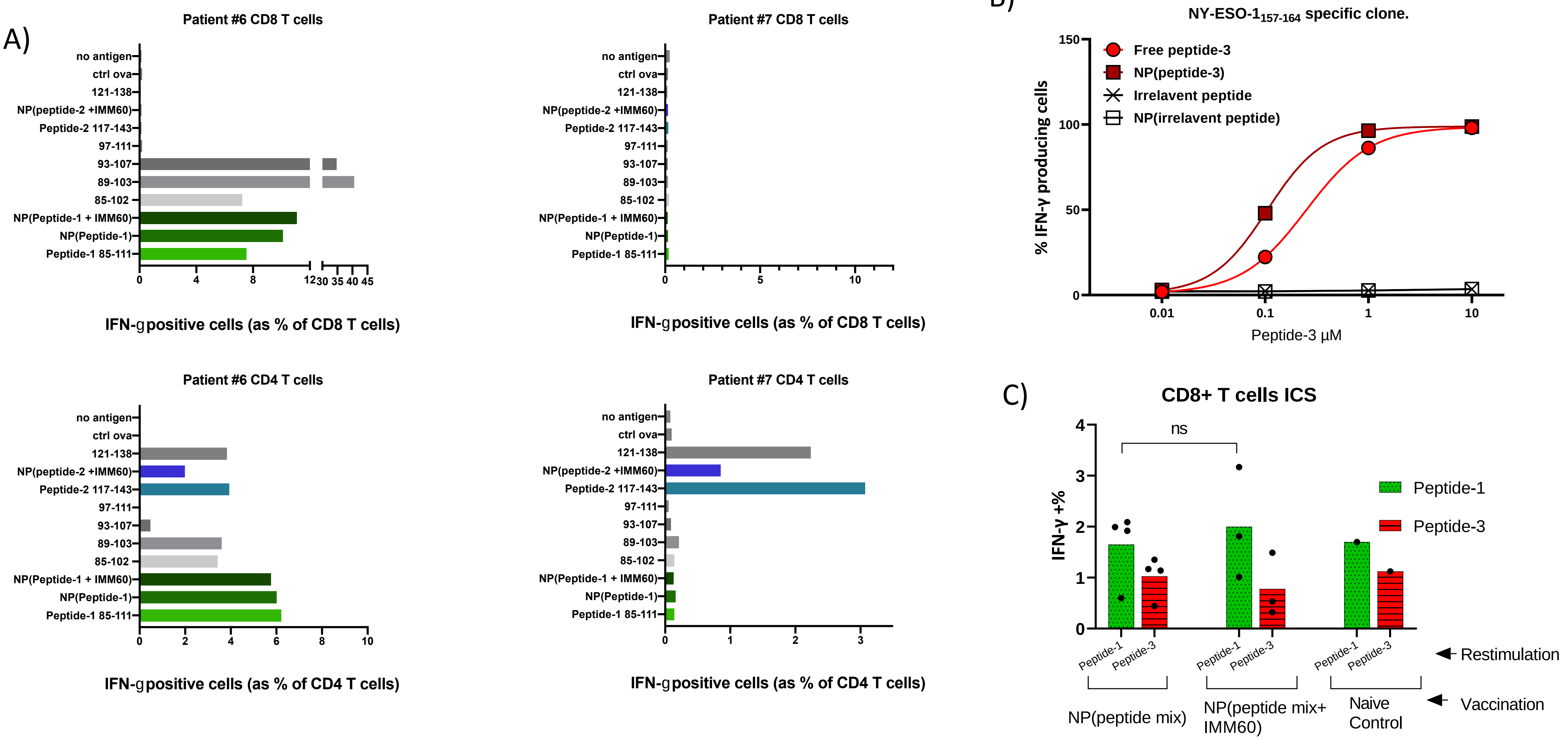

Supplementary figure-3: A) Patient derived PBMCs were stimulated with peptides or nanoparticles which were introduced directly onto the culture and not preloaded onto autologous presenting cells as in figure 3. IFN-γ positive T cells were determined by intracellular staining. **B)** IFN-γ production by CD8+ T cells when peptide-3 either in free form (red circles) or encapsulated in nanoparticles (NP) (red boxes) were delivered to B cells and co incubated with a patient derived CD8 T cell clone (4D8) specific for peptide-3. **C)** C57Bl/6 mice were intravenously injected with 1 mg NPs containing three peptide mix with or without IMM60 or left uninjected. Seven days later splenocytes were isolated and re-stimulated ex-vivo either with peptide 1 or 3 and stained with CD3, CD8 and intracellularly IFN-γ.

Supplementary Figure 4

A)

**Peptide 3**	**157-165 (9mer)**	**SLLMWITQC**
**Peptide 3 LP**	**153-167 (15mer)**	**LQQLSLLMWITQCFL**

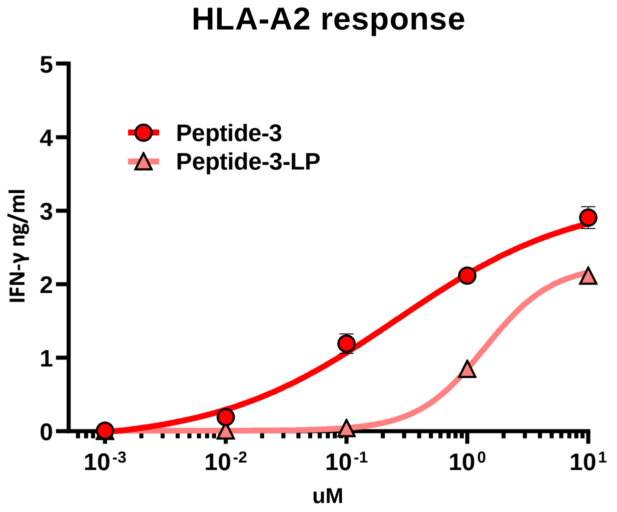

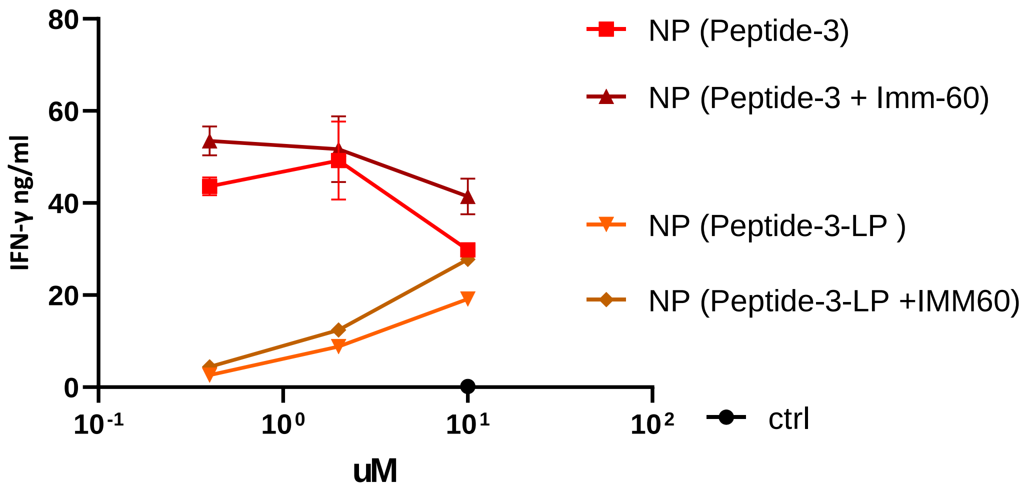

B)

NP(Peptide-3-LP)
2 nmole

NP(Peptide-3-LP)
0.1 nmole

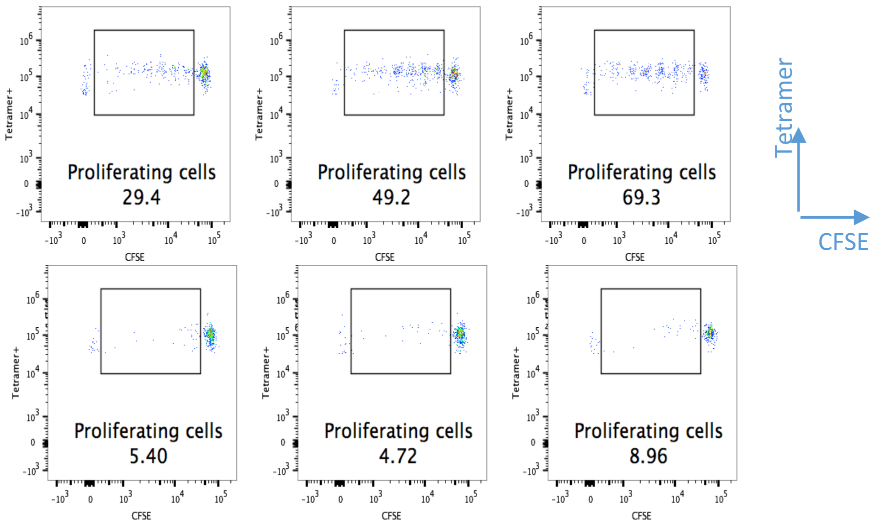

Supplementary figure-4: A) Peptide-3 and Peptide-3-LP sequences are shown in the box (top). Free peptide-3 and peptide-3-LP in solution were compared by means of IFN-γ production by CD8+ T cells transfected with TCR specific for NY-ESO-1 peptide-3 presented by HLA-A2 (Left) . Nanoparticle encapsulated peptide-3 and peptide-3-LP were compared (right). Different donor cells were used in each experiment. **B)** Nanoparticles were injected into F1(HHDxC57BL/6) mice that had received CFSE labelled m1G4 T cells 24 hour before. Proliferation of the m1G4 cell is demonstrated by dilution of the CFSE dye (boxed gate)3 days after injection of the nano particles containing Peptide-3-LP. The amout of peptide injected per animal is shown. The percentage of m1G4 cells that had proliferated is shown below the gate

Supplementary Figure-5

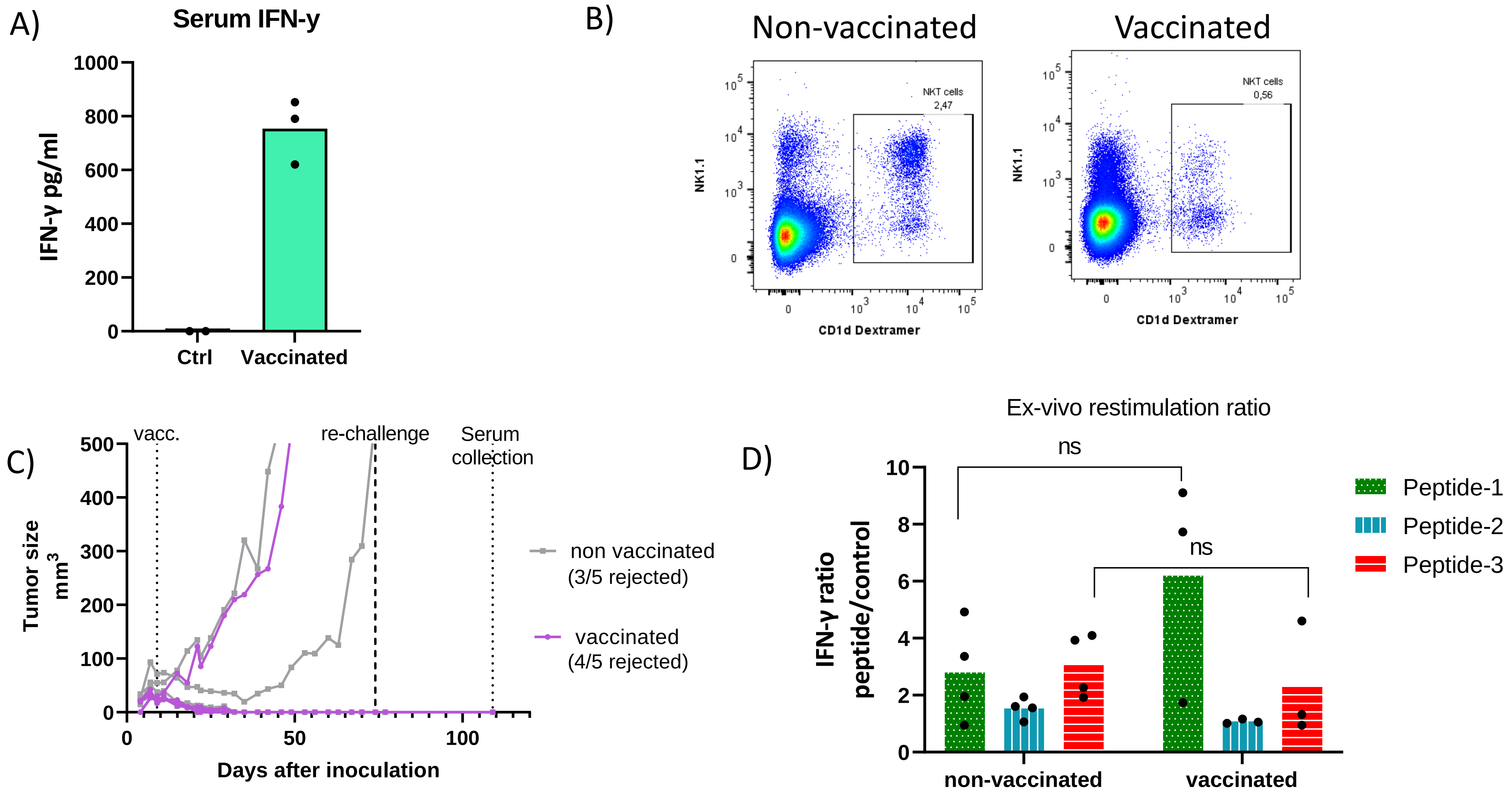

Supplementary figure-5: A) Serum IFN-y levels of the naive homozygous AAD mice 24 hours after iv vaccination with NP(peptide-3+IMM60) indicating an iNKT cell response. **B)** Flow cytometric demonstration of splenic iNKT cells within all CD3+ cells. Left plot, non-vaccinated mice; right plot mice vaccinated with NP(peptide-3+IMM60) one month in advance. iNKT cell numbers decrease and loose their NK1.1 expression upon activation. **C)** Homozygous AAD mice were intravenously injected with 1mg nanoparticles encapsulating three peptide mix and IMM60 or left untreated. 7 months later all mice received 1C12 sarcoma cells expressing full length NY-ESO-1 protein. Vaccinated mice received a booster injection 9 days after tumor inoculation and tumor growths were measured. Remaining mice were rechallanged with sarcoma cells on day 74 and serum was isolated from blood on day 109. **D)** Splenocytes were isolated from the mice shown in C on day 109 and restimulated ex-vivo with three different peptides. Culture supernatants were analysed for IFN-y 72 hours later. The ratio of IFN-y levels of each sample's peptide re-stimulation condition against their control baseline conditions are shown. Each dot represents a mouse.

Supplementary Figure-6

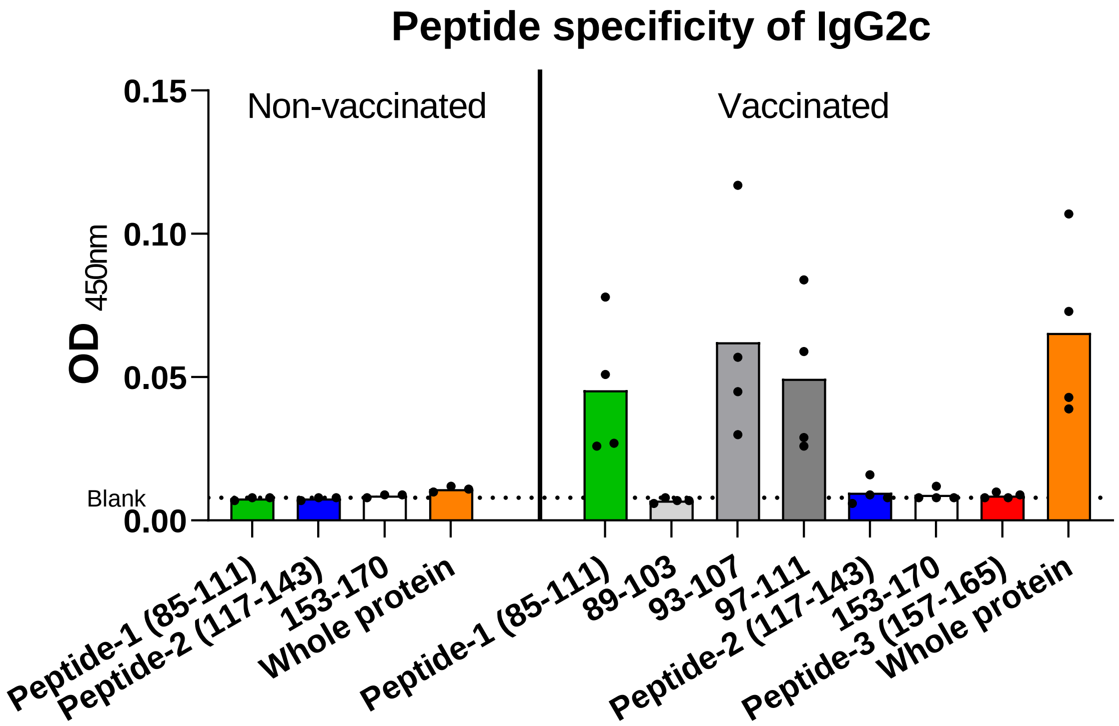

Supplementary figure-6: Peptide specificity of anti-NY-ESO-1 IgG2c antibodies in the sera of AAD mice vaccinated with NP(peptide mix+IMM60) or non-vaccinated and inoculated with 1C12 sarcoma cells expressing full length NY-ESO-1 (Figure-5C). Serum samples were diluted 1/100 before addition on peptide or full protein coated wells, optical density of ELISA wells indicating IgG2c antibody levels are shown. Bars show mean optical density values of all mice, dots represent each mouse.
